# Efficacy of extracellular vesicles as a cell-free therapy in colitis: a systematic review and meta-analysis of animal studies

**DOI:** 10.3389/fphar.2023.1260134

**Published:** 2023-10-26

**Authors:** Jun-Jie Hou, Wei-Wei Li, Xiao-Li Wang, A-Huo Ma, Yue-Hua Qin

**Affiliations:** Department of Gastroenterology, Shaoxing People’s Hospital, Shaoxing, China

**Keywords:** inflammatory bowel disease, extracellular vesicles, colitis, inflammation, therapy

## Abstract

**Background:** Extracellular vesicles (EVs) mediate inflammation, immune responses, gut barrier integrity, and intestinal homeostasis. Recently, the application of EVs in the treatment of inflammatory bowel disease (IBD) has been under intensive focus. Some studies have been conducted in animal models of colitis, while systematic reviews and meta-analyses are lacking. The current study aimed to conduct a systematic review and meta-analysis of studies investigating the efficacy of EVs on IBD.

**Methods:** A systematic retrieval of all studies in PubMed, EMBASE, MEDLINE, Web of Science, and Cochrane Library reported the effects of EVs in the colitis model up to 22 June 2023. The methodological quality was assessed based on SYRCLE’s risk of bias (RoB) tool. Disease activity index (DAI), myeloperoxidase activity (MPO), histopathological score (HS), and inflammatory cytokines (TNF-α, NF-κB, IL-1β, IL-6, and IL-10) were extracted as analysis indicators by Web Plot Digitizer 4.5. A meta-analysis was performed to calculate the standardized mean difference and 95% confidence interval using random-effect models by Review Manager 5.3 and STATA 14.0 software.

**Results:** A total of 21 studies were included in this meta-analysis. Although the heterogeneity between studies and the potential publication bias limits confidence in the extent of the benefit, EV treatment was superior to the control in the colitis evaluation with reduced DAI, HS, MPO activity, and pro-inflammatory cytokines, including TNF-α, NF-κB, IL-1β, and IL-6, while increasing the content of anti-inflammatory cytokine IL-10 (all *p* < 0.05).

**Conclusion:** Our meta-analysis results supported the protective effect of EVs on colitis rodent models based on their potential role in IBD therapy and propelling the field toward clinical studies.

## 1 Introduction

Inflammatory bowel disease (IBD) is a chronic inflammatory intestinal disease including two subtypes, ulcerative colitis (UC) and Crohn’s disease (CD), characterized by chronic relapsing intestinal inflammation and ulcerations (Chang, 2020). It mainly manifests as abdominal pain, diarrhea, bloody stools, and weight loss, profoundly affecting the daily life of patients and increasing the burden on patients, families, and society (Zhao et al., 2021). Hitherto, IBD has affected more than 2.5 million people in Europe and 1 million in the United States, with rising prevalence (Kaplan, 2015; Mak et al., 2020). However, the etiology of IBD is complicated and remains largely unknown. Notably, the potential role of extracellular vesicles (EVs) in intestinal homeostasis and IBD has gained increasing attention (Shen et al., 2022).

EVs have a biomolecular lipid bilayer nanostructure, first described 40 years ago (Pan and Johnstone, 1983), and they are divided into three main subsets, microvesicles (MVs), exosomes, and apoptotic bodies, based on their biogenesis (Colombo et al., 2014; Lötvall et al., 2014). EVs in the gastrointestinal (GI) tract are secreted by immune cells and intestinal epithelial cells (IECs) (van Niel et al., 2001; Robbins and Morelli, 2014) (microbiota-derived EVs cannot be ignored (Deatherage and Cookson, 2012; Brown et al., 2015)) and play a pivotal role in the interaction between IECs (van Niel et al., 2001), endothelial cells (Yamamoto et al., 2015), immune cells (Robbins and Morelli, 2014), and microbiota (Al-Nedawi et al., 2015). These vesicles also regulate the anti-inflammatory reactions (Shen et al., 2022), restoration of vascular and epithelial barrier function (Bui, Mascarenhas, and Sumagin, 2018), recruitment of immune cells (Ocansey et al., 2020), reconstitution of microbiota composition, and the export of cellular metabolites (Toyofuku, Nomura, and Eberl, 2019). Therefore, EVs play a crucial role in maintaining intestinal homeostasis and alleviating intestinal inflammation, showing significant potential in clinical diagnosis and therapy of IBD (Chang et al., 2020; Shen et al., 2022).

Academia and industry have placed high expectations on the therapeutic value of EVs, and they have gradually become a safe, feasible alternative for cell therapy due to significant advantages, which include EVs’ accessibility, high permeability and stability, and low immunogenicity and cytotoxicity (Baek et al., 2019). Accumulating evidence demonstrates the unique advantages of EVs from different cells and tissues in the treatment of multiple diseases. The clinical potential of EVs as biomarkers, therapy, and prognosis assessment for IBD has also been proposed (Chang et al., 2020; Valter et al., 2021; Chen et al., 2022; Nazari et al., 2022; Shen et al., 2022). In inflammatory disease, EV-related therapy is categorized as follows: EVs from stem cells, immune cells, ingesta, and microenvironments as a new remedy or nanocarriers for targeted therapy.

Hitherto, studies on the application of EVs in the treatment of IBD have been carried out in mouse colitis models. Most studies have shown the potential role of EVs in colitis (Yang et al., 2015; Cao L et al., 2019; Choi et al., 2020; Duan et al., 2020; Yang R et al., 2020; Yang Y et al., 2020; Tong et al., 2021a; Tong et al., 2021b; Tolomeo et al., 2021; Wang et al., 2021; Yang et al., 2021; Gan et al., 2022; Gao et al., 2022; Heidari et al., 2022; Kim et al., 2022; Liang et al., 2022; Zhu et al., 2022; Liang et al., 2023; Qian et al., 2023; Tong et al., 2023), while some have put forth contradictory conclusions (Liu L et al., 2021). Also, no systematic review has yet evaluated the efficacy of EVs in mouse models of colitis. Based on the current results, we speculated the protective effect of EVs on colitis. Thus, we conducted this systematic review and meta-analysis to further evaluate the therapeutic efficacy of EVs in colitis models, which might provide a novel strategy for IBD therapy.

## 2 Materials and methods

Our systematic literature review adhered to the Preferred Reporting Items for Systematic Reviews and Meta-Analyses (PRISMA) 2020 guidelines and was registered in the International Prospective Registry of Systematic Reviews (PROSPERO CRD42023430346) on June 2023.

### 2.1 Search strategy

We conducted a comprehensive search in the following electronic databases: PubMed, EMBASE, MEDLINE, Web of Science, and the Cochrane Library of Systematic Reviews from inception to 22 June 2023. The search focused on randomized controlled trials (RCTs) on treatments with EVs from all sources in animal colitis models. The retrieval strategy based on Medical Subject Headings (MeSH) and corresponding free words was modified according to the database, and controlled vocabulary was applied. The detailed search strategy is presented in Supplementary Material S1.

### 2.2 Inclusion and exclusion criteria

The inclusion criteria were as follows: 1) all animal model studies of colitis (all species, all sexes); 2) evaluations of the therapeutic effect of EVs on colitis animal models; 3) all comparators including (but not limited to) placebo, vehicle control, and no treatment; 4) studies reporting indicators for colonic inflammation; 5) all controlled interventional studies; 6) studies published in English.

The exclusion criteria were as follows: works containing 1) comorbidities or defects; 2) non-colitis animal models; 3) EVs that were not administered to animals directly; 4) combination therapy of EVs with other components; 5) instances where the source of EVs has been pre-interfered; 6) EVs that were genetically modified; 7) vitro studies; 8) a lack of endpoints of interest in the study data; 9) incomplete data; 10) no control group or inappropriate comparisons; 11) duplicated studies; 12) no original research (for example, reviews, editorials, letters, and meta-analysis).

### 2.3 Data acquisition

The data related to colitis were extracted as follows: the disease activity index (DAI) represents the severity of colitis; the histopathological score (HS) was used to reflect the pathological changes of colitis. Myeloperoxidase (MPO) activity, tumor necrosis factor-alpha (TNF-α), nuclear factor-kappa B (NF-κB), interleukin-1beta (IL-1β), IL-6, and IL-10 were used to reflect the severity of inflammation. All outcomes were continuous.

In addition, the following data were extracted from all eligible studies: 1) first author; 2) year; 3) location; 4) animal sex, strain; 5) number of each group; 6) modeling method; 7) source of EVs; 8) isolation method; 9) total doses of EVs; 10) times of treatment; 11) delivery route; 12) therapy time; 13) follow-up duration.

### 2.4 Assessment of study quality and bias

The study quality and risk of bias (RoB) were assessed carefully by two independent reviewers using SYRCLE’s RoB tool (Hooijmans et al., 2014) based on the content of the article. The criteria involved six aspects—selection bias, performance bias, detection bias, attrition bias, reporting bias, and other bias—which are further divided into 10 items. Each item consisted of several details and was classified as low, unclear, or high risk of bias. Any discrepancies encountered during the evaluation process were resolved by XL, W.

### 2.5 Data analysis

A routine meta-analysis was performed using Review Manager 5.3 and STATA 14.0 software. For each eligible study, Web Plot Digitizer version 4.5 software was used to extract the data present only in the figures. If the data were not reported or were unclear, we attempted to contact the author via e-mail (maximum of three attempts). To estimate the combined overall effect size, the standardized mean difference (SMD) and 95% confidence interval (CI) were calculated between the treatment and control groups. The heterogeneity of the effect size across the studies was evaluated using *I* ^*2*^ statistics. *I* ^*2*^ = 25%, 50%, and 75% indicated low, moderate, and high heterogeneity, respectively (Higgins and Green, 2011). For *I* ^*2*^ > 50%, a random-effects model was used; otherwise, a fixed-effects model was adopted (Cao et al., 2017). The Z-score was calculated to test the overall effect, with significance set to *p* < 0.05. A sensitivity analysis was performed using STATA version 14.0 to ensure the reliability of the results. The differences in animal experiments were observed with respect to the modeling time and intervention time point. For comparison, the intervention day was defined as the first day of data recording. According to the method described by Vesterinen, H.M.et al. (Vesterinen et al., 2014), if a control group serves multiple experimental groups, the total number of control animals is divided by the number of treatment groups. Subgroup analyses were conducted to explore the effects of potential confounders on the combined effect size estimate. Egger’s statistical tests and funnel plots analysis were used to evaluate the publication bias (Egger et al., 1997). *P* < 0.05 was considered significant for all analyses. A sensitivity analysis examined overall stability.

## 3 Results

### 3.1 Search results

**FIGURE 1:**
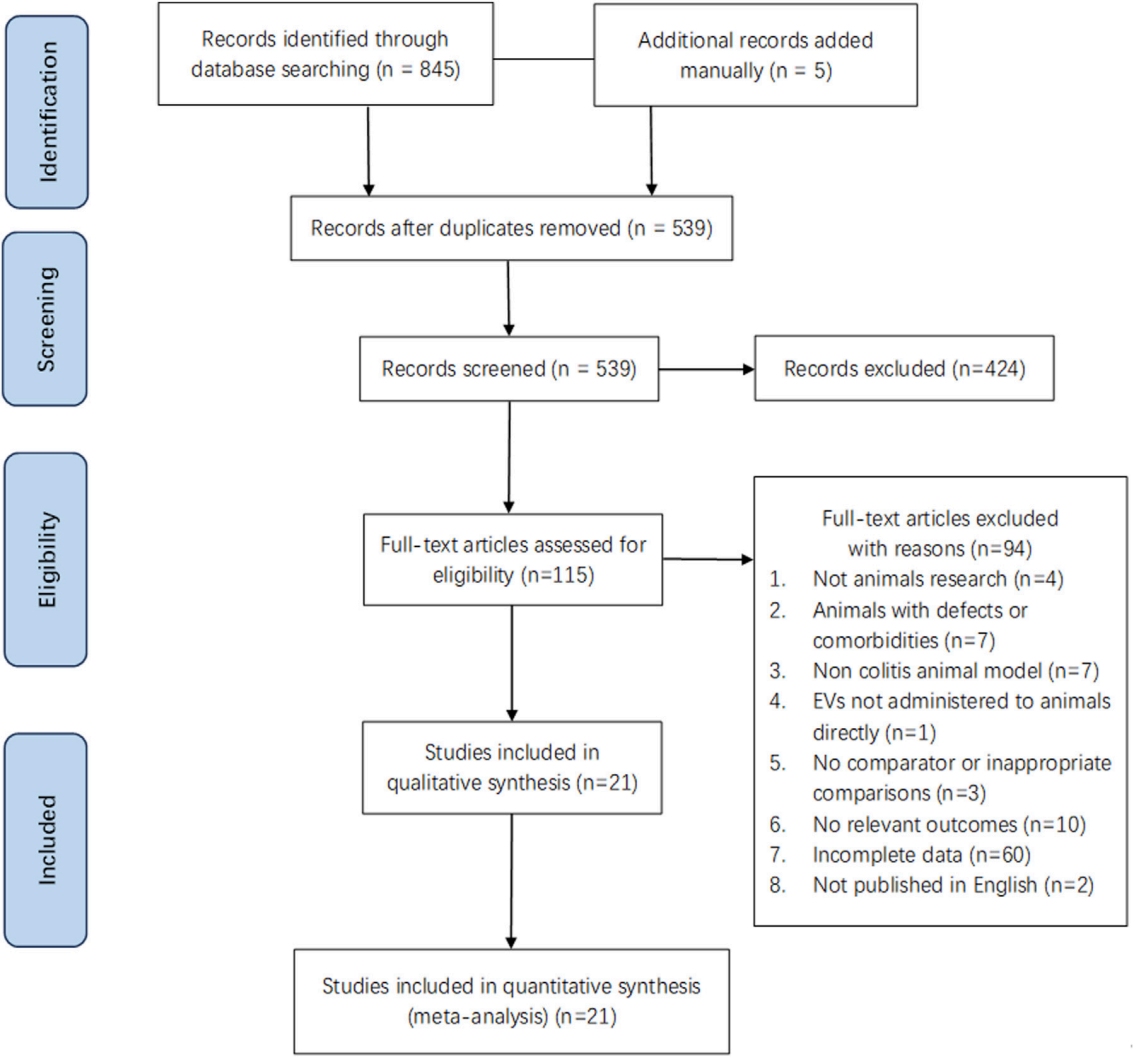
Flow diagram of the study selection.

A total of 850 pieces of literature were retrieved for review (Figure 1). Subsequently, 845 articles were identified from database searches, and 5 additional records were added manually. After removing 311 articles due to duplication, we screened 539 and excluded 424 based on the title and abstract. Then, 115 studies were evaluated through full-text screening, of which 94 were excluded: 4 did not use animal models, 7 due to comorbidities or defects in animals, 7 due to the non-colitis animal model, one because the EVs were not administered to animals directly, 3 because of lack of comparison parameter or inappropriate comparisons, 10 for there being no relevant outcomes, 2 due to being written in a non-English language, and 60 owing to incomplete data. Finally, 21 eligible studies were included in our systematic review and meta-analysis (Yang et al., 2015; Cao L et al., 2019; Choi et al., 2020; Duan et al., 2020; Yang R et al., 2020; Yang Y et al., 2020; Tong et al., 2021a; Tong et al., 2021b; Liu L et al., 2021; Tolomeo et al., 2021; Wang et al., 2021; Yang et al., 2021; Gan et al., 2022; Gao et al., 2022; Heidari et al., 2022; Kim et al., 2022; Liang et al., 2022; Zhu et al., 2022; Liang et al., 2023; Qian et al., 2023; Tong et al., 2023).

### 3.2 Characteristics of the included studies

The baseline characteristics of the included studies are reported in Table 1. A total of 21 studies were included in our review, encompassing 31 comparisons between 2015 and 2023. Among these publications, 2 were in rats, and 19 were in mice. For colitis modeling, 16 studies used dextran sulfate sodium (DSS), 4 used 2,4,6-trinitrobenzene sulfonic acid (TNBS), and 1 study used both. The EVs were derived from various sources, of which there were 11 from mesenchymal stem cells (MSCs), 6 from microbiota, 2 from bovine milk, and 1 each from macrophages and plants. The EVs were isolated through various methods: 16 through ultracentrifugation, 3 used commercial kits, and 1 through ultrafiltration. Moreover, EVs were administered in various units, including absolute protein amount (*n* = 15), particle number (*n* = 1), and based on the weight of the animal (*n* = 5), via intraperitoneal injection (*n* = 7), intravenous injection (N = 5), oral gavage (*n* = 8), and *in situ* injection (*n* = 1). Regarding the therapy time, 12 studies administered the dose before colitis induction, 9 after colitis induction, and 1 used both methods. In terms of treatment frequency, 7 studies involved single administration, while 14 used multiple administrations. The majority of the studies evaluated efficacy at an early stage (<1 week) (*n* = 16), while a few evaluated the efficacy of EVs at a later stage (≥1 week) (*n* = 4), and one study assessed both the early and late stages. We also found that most of the studies were conducted in Asia (*n* = 20), particularly in China (*n* = 17), and primarily in the last 3 years (19/21).

**TABLE 1:** Characteristics of the 21 studies included in the meta-analysis.

Study	Animal	Modeling method	Grouping situation	EVs resource	Isolation method	Total doses of EVs	Treatment frequency	Delivery route	Time of administration	Follow-up duration^*^	Outcome
Cao L et al., 2019, China	BALB/C mice, male	DSS	DSS + NS (*n* = 10)	Mouse BMSCs	Ultracentrifugation	350ug	7	Intraperitoneal injection	After colitis induction	7 Days	DAI, MPO activity, IL-10
			DSS + EVs (*n* = 10)								
Liang, L., et al., 2022, China	C57BL/6 mice, male	DSS	DSS + PBS (*n* = 6)	*Clostridium butyricum*	Ultracentrifugation	150 μg	10	Oral gavage	Before colitis induction	10 Days	DAI
			DSS + *Cb*-EVs (*n* = 6)								
Choi, J.H., et al., 2020, Korea	C57BL/6 mice, male	DSS	DSS + PBS (*n* = 6)	*Lactobacillus paracasei*	Ultracentrifugation	5 mg	1	Oral gavage	After colitis induction	13 Days	DAI
			DSS + *Lp-*EVs (*n* = 6)								
Duan, L., et al., 2020, China	BALB/c mice, male	TNBS	TNBS + PBS (*n* = 8)	hP-MSCs	Ultracentrifugation	200 µg	1	*in situ* injection	After colitis induction	3 Days	DAI, HS, MPO activity
			TNBS + EVs (*n* = 8)								
Yang Y et al., 2020, China	BALB/C mice, female	TNBS	TNBS + PBS (*n* = 10)	*Trichinella spiralis*	Ultracentrifugation	150 µg	3	Intraperitoneal injection	Before colitis induction	6 Days	DAI, HS, MPO activity, TNF-α, IL-1β, IL-10
			TNBS + *Ts*-EVs (*n* = 10)								
Yang, R., et al., ^[28]^, 2020, China	C57BL/6 mice, male	DSS	DSS (*n* = 5)	Mouse BMSCs	Ultracentrifugation	200 μg	1	Intraperitoneal injection	After colitis induction	7 Days	DAI, HS
			DSS + Exos (*n* = 5)								
Wang, Y.X., et al., 2021, China	C57BL/6 mice, male	DSS	DSS (*n* = 6)	Mouse M2 macrophages	Ultracentrifugation	300 μg	6	Caudal vein injection	Before colitis induction	11 Days	DAI, TNF-α, IL-1β, IL-10
			DSS + M2-EVs (*n* = 6)								
Yang, S., et al., 2021, China	BALB/C mice male	TNBS	TNBS + PBS (*n* = 5)	huc-MSCs	ExoQuick-TC exosome isolation reagent	200 μg	1	Intraperitoneal injection	After colitis induction	4 Days	DAI
			TNBS + Exo (*n* = 5)								
Tong et al., 2021a, China	C57BL/6J mice, male	DSS	DSS + PBS	*Lactobacillus rhamnosus* GG	Ultracentrifugation	1.2 mg/kg	1	Oral gavage	Before colitis induction	22 Days	NF-κB, IL-6
			DSS + *LGG*-EVs								
Kim, H.J., et al., 2022, Korea	C57BL/6 mice, male	DSS	DSS (*n* = 5)	*Giardia lamblia*	Centrifugation ExoQuick TC Kit	30 µg	3	Intraperitoneal injection	Before colitis induction	43 Days	DAI, HS
			DSS + *GI*-EVs (*n* = 5)								
Heidari, N., et al., 2022, Iran	C57BL/6 mice, female	DSS	DSS + PBS (*n* = 7)	huc-MSCs	EXOCIB kit	300 μg	3	Intraperitoneal injection	After colitis induction	7 Days	DAI, HS
			DSS + MSC-Exos (*n* = 7)								
Tong et al., 2021a, China	C57BL/6 mice, male	DSS	DSS + PBS	Bovine milk	Ultracentrifugation		25	Oral gavage	Before colitis induction	24 Days	MPO activity, TNF-α, NF-κB, IL-1β, IL-6
			DSS + mEVs-L			0.6 mg/kg/day×25 day					
			DSS + mEVs-M			1.8 mg/kg/day×25 day					
			DSS + mEVs-H			3 mg/kg/day×25 day					
Yang, J., et al., 2015, China	SD rats, male	TNBS	TNBS + PBS (*n* = 10)	Rat BMSCs	Ultracentrifugation		1	Caudal vein injection	After colitis induction	7 Days	DAI, MPO activity, NF-κB, IL-1β, IL-10
			TNBS + EVs-L (*n* = 10)			50 ug					
			TNBS + EVs-M (*n* = 10)			100 ug					
			TNBS + EVs-H (*n* = 10)			200 ug					
Liu L et al., 2021, China	C57BL/6 mice, male	DSS	DSS + PBS (*n* = 8–12)	*Fusobacterium nucleatum*	Ultracentrifugation		10	Oral gavage	Before colitis induction	10 Days	DAI
			DSS + low *Fn*-EVs (n = 8–12)			100 μg					
			DSS + high *Fn*-EVs (*n* = 8–12)			500 μg					
Tolomeo, A.M., et al., 2021, France	C57BL/6 mice	DSS	DSS + PBS (*n* = 5)	Mouse BMSCs	Ultrafiltration	3.00E+09	3	Intraperitoneal injection	Before colitis induction	6 Days	DAI, HS
			DSS + nEVs (*n* = 5)								
Tong, L., et al., 2023, China	C57BL/6 mice	DSS (acute)	DSS + fresh water	Bovine milk	Ultracentrifugation		7	Oral gavage	Before colitis induction	17 Days	DAI
			DSS + mEVs-L			0.6 mg/kg×7 day					
			DSS + mEVs-H			3.0 mg/kg×7 day					
		DSS (chronic)	DSS + fresh water							33 Days	DAI, IL-10
			DSS + mEVs-L			0.6 mg/kg×7 day					
			DSS + mEVs-H			3.0 mg/kg×7 day					
Liang, X., et al., 2023, China	C57BL/6 mice, Male	DSS	DSS + PBS	huc-MSCs	ExoQuick-TC exosome isolation reagent	900ug	3	Intraperitoneal injection	Before colitis induction	6 Days	DAI, MPO activity, HS
			DSS + Exos								
	BABL/c mice, Male	TNBS	TNBS + PBS			600 ug	2		After colitis induction	12 Days	
			TNBS + Exos								
Qian, W., et al., 2023, China	C57BL/6J mice, Male	DSS	DSS + Saline (*n* = 5)	Mouse ASCs	Ultracentrifugation	200 ug	1	Caudal vein injection	After colitis induction	7 Days	DAI, HS, TNF-α, IL-1β, IL-6, IL-10
			DSS + Exo (*n* = 5)								
Zhu, F., et al., 2022, China	SD rats, male	DSS	DSS + PBS (*n* = 6)	Rat BMSCs	Ultracentrifugation	Unknown (100 ug/mL)	2	Caudal vein injection	After colitis induction	6 Days	DAI
			DSS + Exos (*n* = 6)								
Gan, J., et al., 2022, China	C57 mice, Male	DSS	DSS + Saline (*n* = 6)	huc-MSCs	Ultracentrifugation	20 mg/kg	3	Oral gavage	After colitis induction	8 Days	DAI, MPO activity, HS
			DSS + Exo (*n* = 6)								
Gao, C.F., et al., 2022, China	ICR mice (female and male)	DSS (acute)	DSS + PBS	Turmeric	Ultracentrifugation	10 mg/kg	12	Oral gavage	After colitis induction	12 Days	DAI, MPO activity, TNF-α, IL-1β, IL-10
			DSS + TNVs								
		DSS (chronic)	DSS + PBS			Unknown	3			56 Days	DAI, MPO activity
			DSS + TNVs								

BMSCs, bone marrow mesenchymal stem cells; BMDMs, bone marrow-derived macrophages; DAI, disease activity index; DC, differential centrifugation; DSS, dextran sulfate sodium; TNBS, trinitrobenzene sulfonic acid; HS, histopathological score; hP-MSCs, human placental mesenchymal stem cells; huc-MSCs, human umbilical cord mesenchymal stem cells; MSCs, mesenchymal stem cells; NS, normal saline; TNVs, turmeric-derived nanovesicles.

### 3.3 Risk of bias within studies

The SYRCLE’s RoB tool was used to assess the quality of the included studies. The results of methodological quality are presented in Table 2. Generally, none of the studies presented a low risk of bias. While most studies (12/21, 57.14%) reported baseline characteristics of animals, 1/21 mentioned random sequence generation. Also, the risk of allocation concealment could not be confirmed because none of the studies offered complete information. Moreover, most studies did not describe the methods for random housing, random outcome assessment, and blinding of the researcher and evaluator. The RoB assessment revealed a low risk of 52.86%, an unclear risk of 46.19%, and a high risk of 0.95% among these studies. Due to the lack of published protocols, it was challenging to determine any selective reporting bias in the studies; however, no other sources of bias were found.

**TABLE 2:** SYRCLE’s risk of bias tool for each experimental animal study.

ItemStudy	Sequence generation	Baseline characteristics	Allocation concealment	Random housing	Blinding (study team)	Random outcome assessment	Blinding (outcome assessors)	Incomplete outcome data	Selective outcome reporting	Other bias
	Selection bias			Performance bias		Detection bias		Attrition bias	Reporting bias	Other
Cao L et al., 2019, China	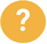	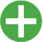	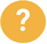	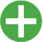	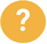	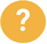	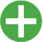	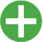	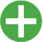	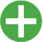
Liang, L., et al., 2022, China	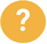	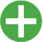	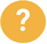	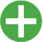	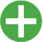	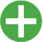	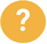	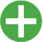	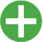	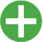
Choi, J.H., et al., 2020, Korea	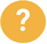	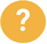	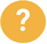	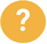	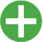	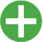	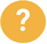	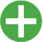	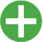	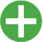
Duan, L., et al., 2020, China	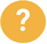	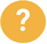	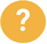	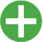	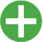	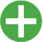	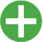	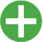	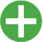	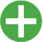
Yang Y et al., 2020, China	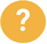	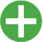	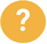	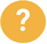	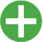	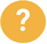	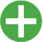	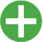	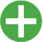	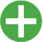
Yang R et al., 2020, China	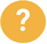	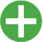	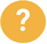	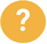	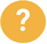	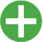	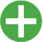	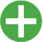	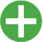	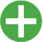
Wang, Y.X., et al., 2021, China	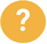	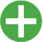	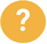	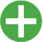	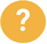	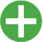	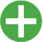	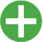	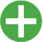	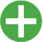
Yang, S., et al., 2021, China	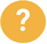	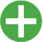	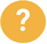	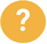	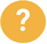	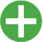	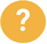	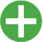	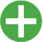	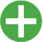
Tong et al., 2021a, China	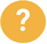	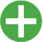	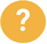	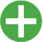	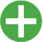	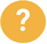	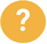	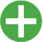	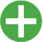	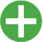
Kim, H.J., et al., 2022, Korea	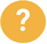	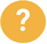	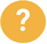	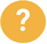	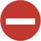	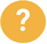	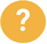	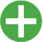	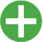	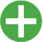
Heidari, N., et al., 2022, Iran	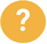	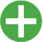	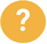	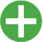	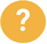	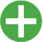	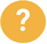	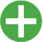	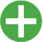	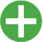
Tong et al., 2021a, China	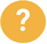	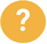	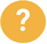	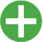	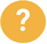	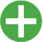	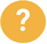	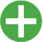	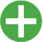	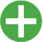
Yang, J., et al., 2015, China	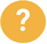	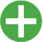	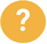	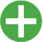	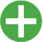	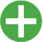	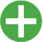	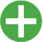	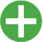	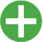
Liu L et al., 2021, China	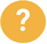	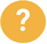	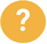	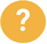	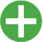	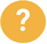	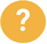	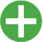	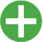	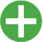
Tolomeo, A.M., et al., 2021, France	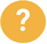	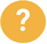	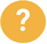	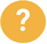	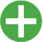	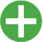	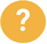	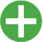	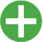	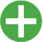
Tong, L., et al., 2023, China	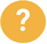	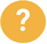	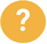	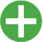	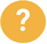	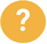	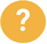	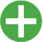	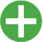	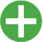
Liang, X., et al., 2023, China	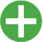	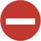	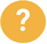	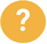	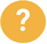	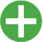	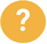	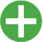	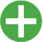	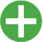
Qian, W., et al., 2023, China	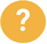	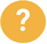	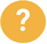	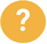	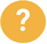	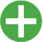	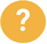	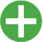	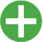	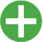
Zhu, F., et al., 2022, China	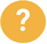	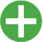	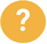	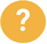	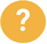	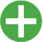	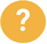	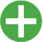	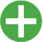	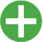
Gan, J., et al., 2022, China	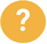	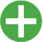	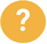	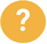	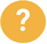	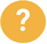	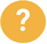	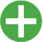	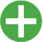	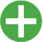
Gao, C.F., et al., 2022, China	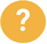	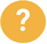	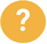	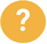	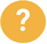	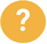	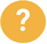	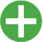	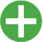	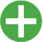

### 3.4 Efficacy evaluation

#### 3.4.1 DAI

**FIGURE 2:**
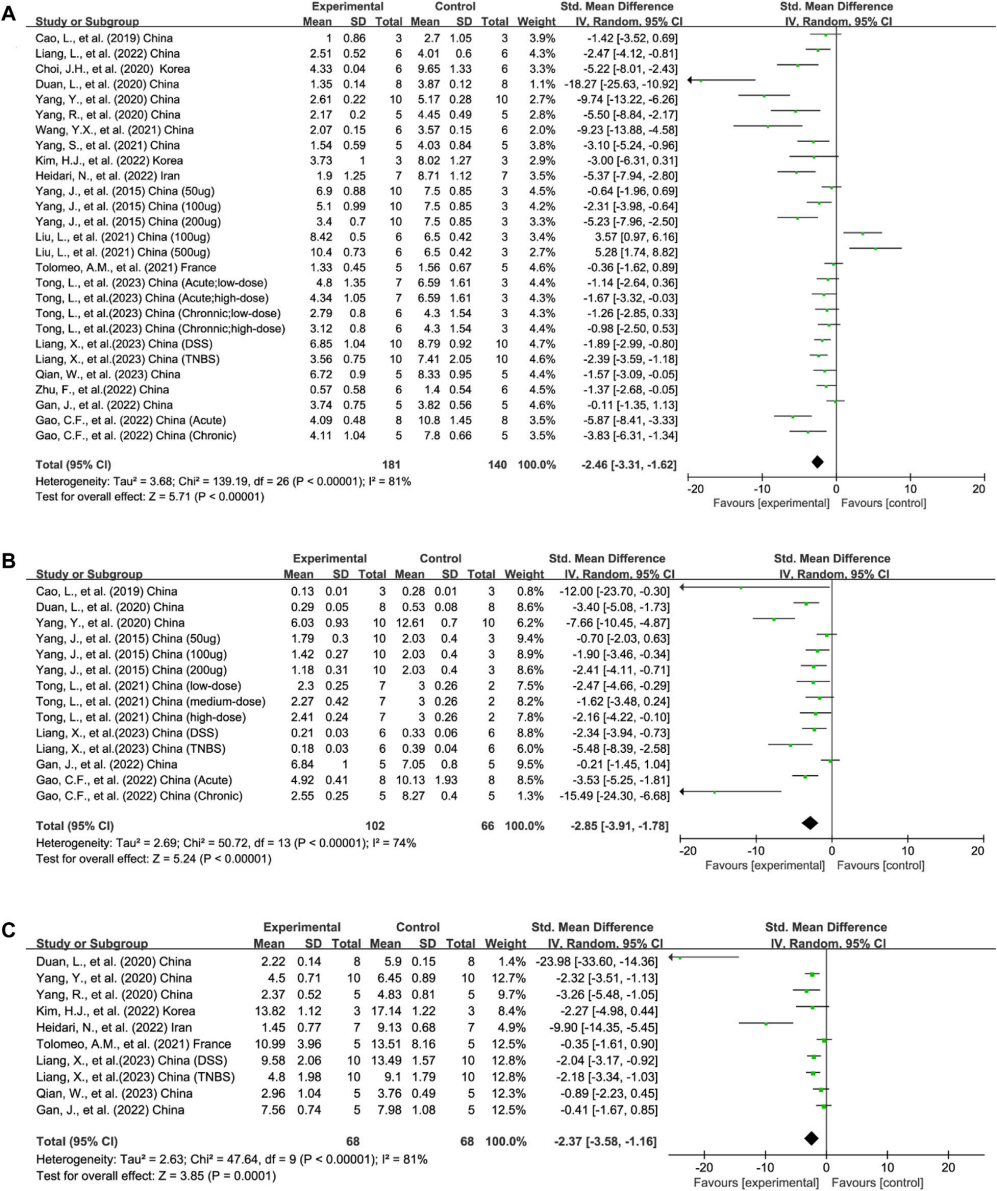
Forest plots summarize the effect of EVs on **(A)** DAI, **(B)** MPO activity, and **(C)** HS in animal models of colitis.

Disease severity was reported in 19/21 publications as DAI (Yang et al., 2015; Cao L et al., 2019; Choi et al., 2020; Duan et al., 2020; Yang R et al., 2020; Yang Y et al., 2020; Liu L et al., 2021; Tolomeo et al., 2021; Wang et al., 2021; Yang et al., 2021; Gan et al., 2022; Gao et al., 2022; Heidari et al., 2022; Kim et al., 2022; Liang et al., 2022; Zhu et al., 2022; Liang et al., 2023; Qian et al., 2023; Tong et al., 2023). It is scored by evaluating the extent of body weight loss, stool consistency, and stool bleeding of mice and examined the effects of EVs across 27 experiments (total *n* = 321; *n* = 181 experimental vs. 140 in control groups). The EVs significantly reduced DAI compared to the control group (*n* = 321; SMD = −2.46; 95% CI: −3.31 to −1.62; *p* < 0.05; *I* ^*2*^ = 81%) (Figure 2A).

#### 3.4.2 MPO activity

MPO is a marker of neutrophil activation and a clinical index for IBD. MPO activity was applied in 14 experiments across 8/21 publications to reflect the degree of neutrophil infiltration (total *n* = 168; *n* = 102 experimental vs. 66 in control groups) (Yang et al., 2015; Cao L et al., 2019; Duan et al., 2020; Yang Y et al., 2020; Gan et al., 2022; Gao et al., 2022; Liang et al., 2023; Tong et al., 2023). The lower MPO activity in the experimental group than in the control group favored the efficacy of EVs on colitis (*n* = 168; SMD = −2.85; 95% CI: −3.91 to −1.78; *p* < 0.05; *I* ^*2*^ = 74%) (Figure 2B).

#### 3.4.3 HS

HS was adopted in 9/21 publications containing 10 experiments measuring the severity of inflammation based on the extent of inflammation, inflammatory cell infiltration, crypt damage, and loss of goblet cells (total *n* = 136; *n* = 68 experimental vs. 68 in control groups) (Duan et al., 2020; Yang R et al., 2020; Yang Y et al., 2020; Tolomeo et al., 2021; Gan et al., 2022; Heidari et al., 2022; Kim et al., 2022; Liang et al., 2023; Qian et al., 2023). Compared to the control group, the HS of the EV experimental group decreased significantly (n = 136; SMD = −2.37; 95% CI: −3.58, −1.16; *p* < 0.05; *I* ^*2*^ = 81%) (Figure 2C).

#### 3.4.4 Inflammatory mediators

##### 3.4.4.1 TNF-α

**FIGURE 3:**
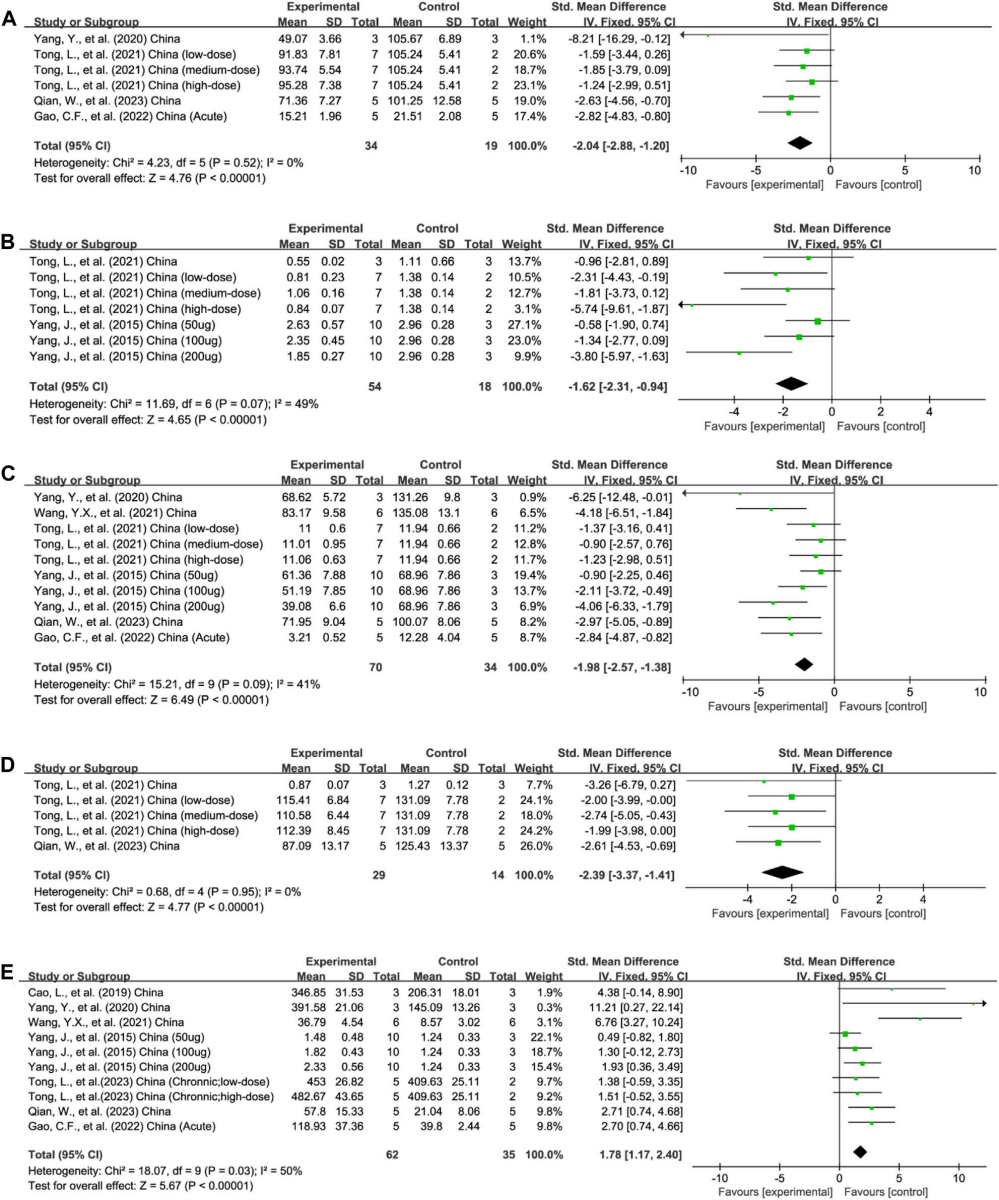
Forest plots summarize the effect of EVs on **(A)** TNF-α, **(B)** NF-κB, and **(C)** IL-1β, **(D)** IL-6, and **(E)** IL-10 in animal models of colitis.

Four studies reported TNF-α outcomes in six experiments (total *n* = 53; *n* = 34 experimental vs. 19 in control groups) (Yang Y et al., 2020; Tong et al., 2021a; Gao et al., 2022; Qian et al., 2023). The protein levels of colonic TNF-α were decreased after EV therapy (*n* = 53; SMD = −2.04; 95% CI: −2.88 to −1.20; *p* < 0.05; *I* ^*2*^ = 0%) (Figure 3A).

##### 3.4.4.2 NF-κB

The protein levels of colonic NF-κB were reported in 3/21 studies encompassing 7 experiments (total n = 72; *n* = 54 experimental vs. 18 in control groups) (Yang et al., 2015; Tong et al., 2021b; Tong et al., 2023). The colonic NF-κB protein levels were downregulated after EV therapy (n = 172; SMD = −1.62; 95% CI: −2.31 to −0.94; *p* < 0.05; *I* ^*2*^ = 49%) (Figure 3B).

##### 3.4.4.3 IL-1β

In total, 6/21 studies, involving 10 experiments, evaluated the content of pro-inflammatory IL-1β in the colon tissue (total *n* = 104; *n* = 70 experimental vs. 34 in control groups) (Yang et al., 2015; Yang Y et al., 2020; Tong et al., 2021b; Wang et al., 2021; Gao et al., 2022; Qian et al., 2023). EV treatment decreased the content of IL-1β (*n* = 104; SMD = −1.98; 95% CI: −2.57 to −1.38; *p* < 0.05; *I* ^*2*^ = 41%) (Figure 3C).

##### 3.4.4.4 IL-6

Five experiments across three studies assessed the level of IL-6 in colon tissue (total *n* = 43; *n* = 29 experimental vs. 14 in control groups) (Tong et al., 2021a; Tong et al., 2021b; Qian et al., 2023). EV treatment reduced the pro-inflammatory IL-6 concentration (n = 43; SMD = −2.39; 95% CI: −3.37 to −.41; *p* < 0.05; *I* ^*2*^ = 0%) (Figure 3D).

##### 3.4.4.5 IL-10

In total, 7/21 studies with 10 experiments assessed the concentration of anti-inflammatory IL-10 levels in the colon tissue (total *n* = 97; n = 62 experimental vs. 35 in control groups) (Yang et al., 2015; Cao L et al., 2019; Yang Y et al., 2020; Wang et al., 2021; Gao et al., 2022; Qian et al., 2023; Tong et al., 2023). Animals receiving EV intervention showed a higher IL-10 concentration (n = 97; SMD = 1.78; 95% CI: 1.17–2.40; *p* < 0.05; *I* ^*2*^ = 50%) (Figure 3E).

### 3.5 Subgroup analysis

Based on the results of the analysis described above, we analyzed DAI (Supplementary Material S2, Supplementary Table S1), MPO activity (Supplementary Material S2, Supplementary Table S2), HS (Supplementary Material S2, Supplementary Table S3), IL-1β (Supplementary Material S2, Supplementary Table S4), and IL-10 (Supplementary Material S2, Supplementary Table S5) in different subgroups stratified according to the following variables: animal species (rat and mouse), animal model (DSS and TNBS), EV source (MSCs, microbiota, milk, macrophages, and plants), total doses of EVs (<200 mg, ≥200 mg, and unknown dose), time of administration (before and after colitis induction), delivery route (intraperitoneal injection, intravenous injection, oral gavage, and *in situ* injection), treatment frequency (single and multiple administrations), follow-up duration (<1 week and ≥1 week), and EVs’ isolation method (ultracentrifugation, commercial kits, and ultrafiltration). The detailed figures are described in Supplementary Material S3.

#### 3.5.1 DAI

The heterogeneity was significantly reduced in milk-derived EVs (*I* ^*2*^ = 0%), plant-derived EVs (*I* ^*2*^ = 22%), and later stage effect (*I* ^*2*^ = 0%) but was significant in the remaining subgroups. A significant difference was observed in the effect size in the model type (*p* < 0.05), EV source (*p* < 0.05), total doses of EVs (*p* < 0.05), delivery route (*p* < 0.05), and EV isolation method (*p* < 0.05), while animal species (*p* = 0.55), therapy time (*p* = 0.21), treatment frequency (*p* = 0.08), and follow-up duration (*p* = 0.07) were not. Furthermore, the EVs improved the DAI more in experiments involving mice, TNBS models, ≥200 mg doses, medication after colitis induction, single administration, and early-stage effect than in other groups. Commercial kits displayed both low heterogeneity (*I* ^*2*^ = 38%) and better efficacy. Regarding EV source and delivery route, the *in situ* injection and macrophage-derived EVs presented better results; however, both parameters were addressed in only one study. (Supplementary Material S3, Supplementary Figures S1A-I).

#### 3.5.2 MPO activity

The treatment of rats with EVs (*I* ^*2*^ = 27%), derived from milk (*I* ^*2*^ = 0%), ≥200 mg dose (*I* ^*2*^ = 28%), and intravenous administration (*I* ^*2*^ = 27%), provided more stable outcomes compared to other groups. No differences were observed in the effect size among the model type (*p* = 0.46), total doses of EVs (*p* = 0.71), therapy time (*p* = 0.81), delivery route (*p* = 0.07), treatment frequency (*p* = 0.15), follow-up duration (*p* = 0.97), and EV isolation method (*p* = 0.57). TNBS-induced colitis, <200 mg dose of EVs, the use of EVs before colitis induction, intraperitoneal injection, multiple therapies, and commercial kits showed better efficacy in MPO activity. In the subgroup of follow-up duration, early- and late-stages produced a similar effect. The animal species (*p* < 0.05) and the source of EVs (*p* < 0.05) may result in differential effects. In addition, the experiments involving mice and microbiota-derived EVs significantly attenuate MPO activity. (Supplementary Material S3, Supplementary Figures S2A-I).

#### 3.5.3 HS

Low heterogeneity was observed in microbiota-derived EVs (*I* ^*2*^ = 0%), <200 mg doses of EVs (*I* ^*2*^ = 0%), and therapy before colitis induction (*I* ^*2*^ = 0%), but high heterogeneity was found in other subgroups. In addition, model type (*p* = 0.18), EVs source (*p* = 0.80), time of administration (*p* = 0.51), treatment frequency (*p* = 0.15), and follow-up duration (*p* = 0.93) showed no significant differences in the effect size, while significant differences were observed in the dose (*p* < 0.05), delivery route (*p* < 0.05), and isolation method (*p* < 0.05) of EVs. Among these, the TNBS-induced colitis, MSCs-derived EVs, intervention after colitis induction, single administration, and early-stage effect of EVs, ≥200 mg dose of EVs, and commercial kits showed an improved HS. Oral gavage exhibited an obvious improvement over other methods; however, only one study considered this comparison. (Supplementary Material S3, Supplementary Figures S3A-H).

#### 3.5.4 IL-1β

Studies on the effect of EVs on IL-1β showed low heterogeneity (*I* ^*2*^ = 41%). Moreover, the subgroup analysis revealed that the animal species (*p* = 0.75), modeling reagent (*p* = 0.95), EVs source (*p* = 0.07), administration time (*p* = 0.47), delivery route (*p* = 0.17), medication frequency (*p* = 0.83), and observation period (*p* = 0.05) showed no significant differences in the effect size, while the dosage of EVs (*p* < 0.05) displayed significant differences that ≥200 mg dose downregulated the IL-1β levels. Furthermore, the experiments involving mice, medication after colitis induction, single administration, intravenous EVs, and early-effect of EVs appeared more effective for macrophage-derived EVs, but this was reported by only one study. (Supplementary Material S3, Supplementary Figures S4A-H).

#### 3.5.5 IL-10

The subgroup analysis suggested that the model type might be the source of heterogeneity; the DSS-induced (*I* ^*2*^ = 42%) and TNBS-induced (*I* ^*2*^ = 42%) groups showed reduced heterogeneity. It also revealed significant differences in the effect size (*p* < 0.05), with DSS modeling showing a stronger ability to elevate IL-10 levels. Additionally, the heterogeneity of the studies on mice (*I* ^*2*^ = 31%) was low in MSCs- (*I* ^*2*^ = 29%) and milk-derived (*I* ^*2*^ = 0%) EVs, an unknown dose of EVs (based on the weight of the animal) (*I* ^*2*^ = 0%), administration after colitis induction (*I* ^*2*^ = 28%), single administration (*I* ^*2*^ = 25%), the delivery method of intraperitoneal injection (*I* ^*2*^ = 22%), oral gavage (*I* ^*2*^ = 0%), and late-staged potency (*I* ^*2*^ = 0%), yet other groups remains high. Significant differences in effect size were detected with respect to EV source (*p* < 0.05), wherein EVs derived from bacteria had high IL-10 levels. However, the animal species (*p* = 0.27), therapeutic dose (*p* = 0.10), treatment time (*p* = 0.35), delivery method (*p* = 0.22), therapy frequency (*p* = 0.08), and follow-up duration (*p* = 0.61) showed no significant differences. Among these, the studies on mice, ≥200 mg dose treatment, medication before colitis, intraperitoneal injection of EVs, multiple administration, and early effect of EVs elevated the IL-10 levels. (Supplementary Material S3, Supplementary Figures S5A-H).

### 3.6 Publication bias

**FIGURE 4:**
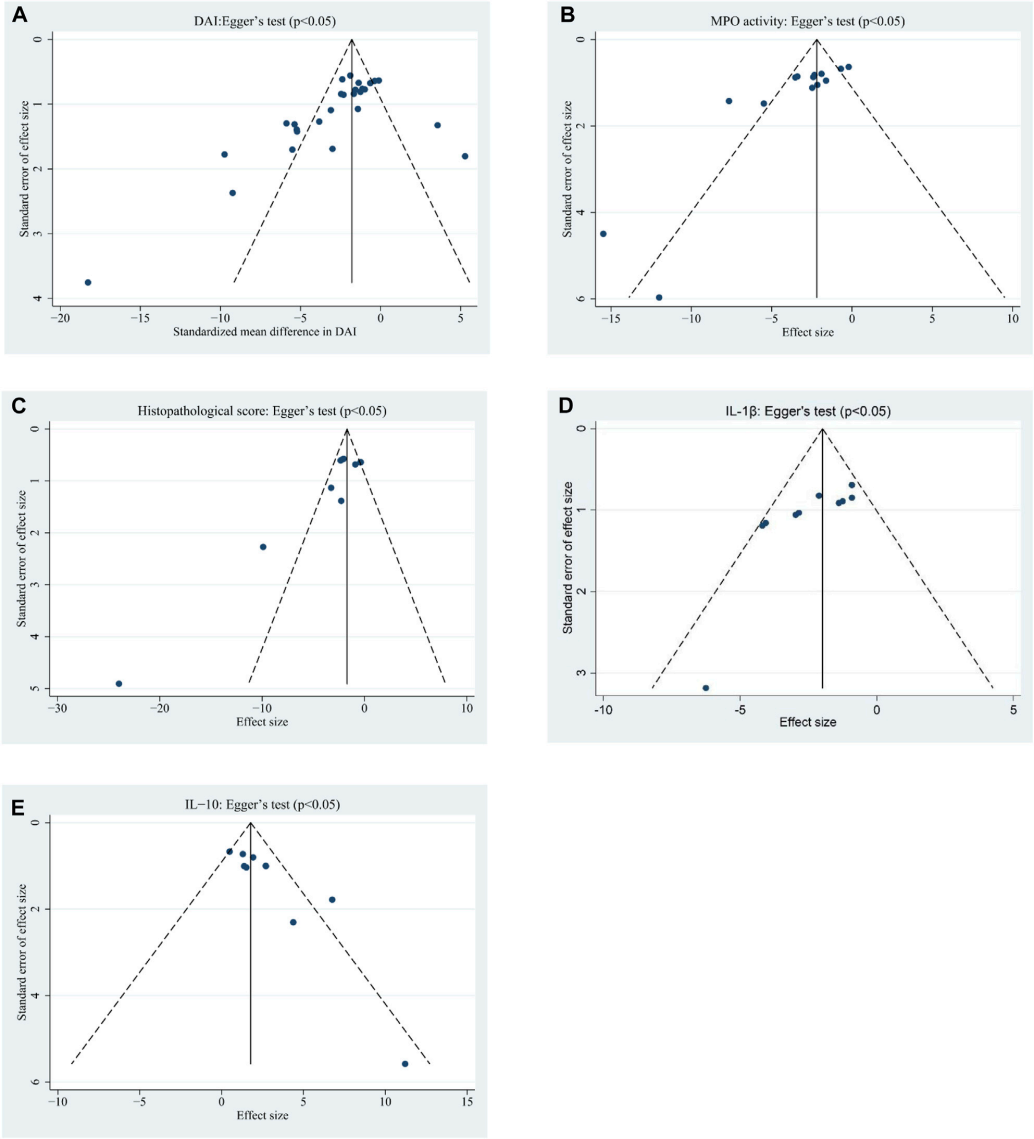
Funnel plots assessing publication bias studies investigating the effects of EVs on **(A)** DAI, **(B)** MPO activity, **(C)** HS, **(D)** IL-1β, and **(E)** IL-10 in animal models of colitis.

For indicators that included nine or more studies, Egger’s statistical test and funnel plot analysis were used to evaluate publication bias. Considering that the TNF-a, NF-κB, and IL-6 indexes were carried out in <10 experiments, funnel plot analysis and Egger’s tests for publication bias were not performed. The visual inspection of funnel plots indicated asymmetry for DAI (Figure 4A), MPO activity (Figure 4B), HS (Figure 4C), IL-1β (Figure 4D), and IL-10 (Figure 4E). Egger’s test for publication bias showed significant effects for DAI (*p* < 0.05), MPO activity (*p* < 0.05), HS (*p* < 0.05), IL-10 (*p* < 0.05), and IL-1β (*p* < 0.05).

### 3.7 Sensitivity analysis

**FIGURE 5:**
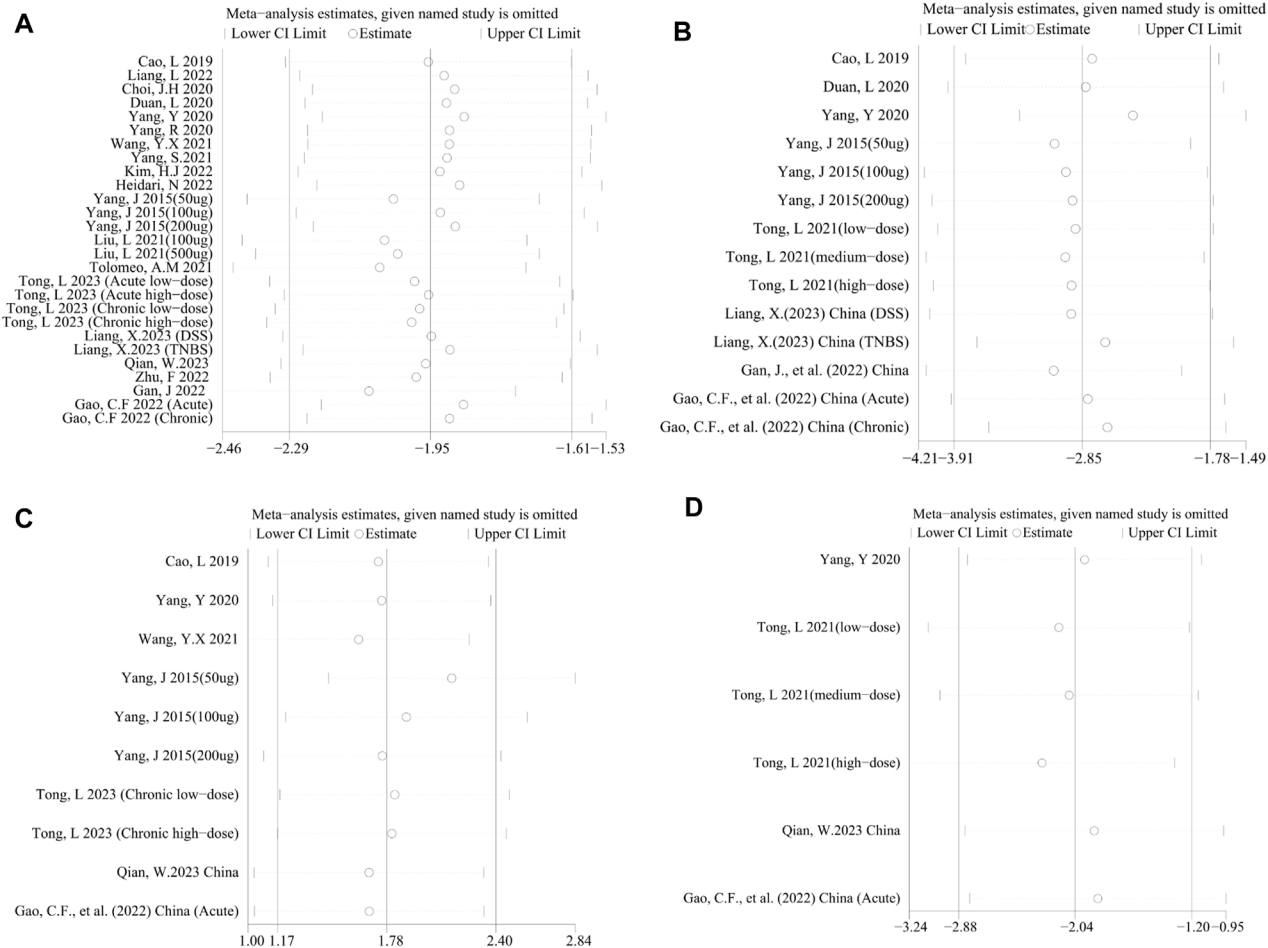
Sensitivity analysis of the studies included in **(A)** DAI, **(B)** MPO activity, **(C)** HS, and **(D)** TNF-α.

**FIGURE 6:**
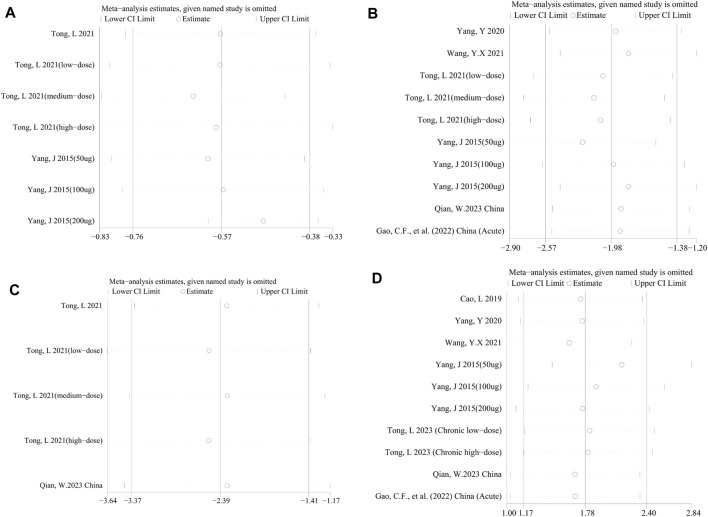
Sensitivity analysis of the studies included in **(A)** NF-κB, **(B)** IL-1β, **(C)** IL-6, and **(D)** IL-10.

Considering significant heterogeneity, we performed a sensitivity analysis to determine the stability of results by sequentially excluding each study. Consequently, the pooled SMDs of DAI (Figure 5A), MPO activity (Figure 5B), HS (Figure 5C), TNF-α (Figure 5D), NF-κB (Figure 6A), IL-1β (Figure 6B), IL-6 (Figure 6C), and IL-10 (Figure 6D) were affected by any of the studies.

## 4 Discussion

The present study aimed to determine the efficacy of EVs in reducing intestinal inflammation, formulate additional clinical studies, and explore novel treatment strategies. Although the studies were heterogeneous, general trends were identified. We found that Evs effectively improved DAI, HS, and MPO activity, upregulated the expression of anti-inflammatory IL-10, and downregulated the expression of pro-inflammatory factors, including TNF-α, NF-κB, IL-1β, and IL-6 in colitis animals. These findings support the efficacy of Evs in colitis and provide clues for the potential development of human clinical trials on Evs and novel treatments for IBD.

SYRCLE’s RoB tool was used to assess the methodological quality of the included 21 studies in this meta-analysis. We found that the methods of most studies lacked clear methodological descriptions and had a potential bias due to insufficient randomization and lack of blinding. Moreover, no standard methods have been developed for EV administration in animal models of colitis. Therefore, a comprehensive description of the specific methods for the standardized characterization of EVs is essential.

Evs facilitate the transport of various molecules over long distances inside the body to mediate homeostasis and the development of IBD through multiple mechanisms (Valter et al., 2021; Shen et al., 2022). In the intestinal microenvironment of patients with active IBD, the recruitment of innate immune cells, such as neutrophils, macrophages, monocytes, dendritic cells (DCs), and T-cells is increased, which produces EVs that mediate immune responses and inflammation; this phenomenon is closely related to IBD pathogenesis. Additionally, EVs trigger the NF-κB signaling pathway to improve intestinal barrier function (Nata et al., 2013). The increased release of EVs containing high levels of TGF-β1 (Jiang et al., 2016) and Annexin-1 (a calcium-dependent phospholipid-binding protein known for its anti-inflammatory properties) (Leoni et al., 2015; Sheikh and Solito, 2018) also accelerates barrier restitution. Furthermore, EVs interact with the bacterial surface and effectuate bacterial aggregation (van Bergenhenegouwen et al., 2014), while EVs nucleic acids participate in microbial structure conformation (Shen et al., 2022). EVs from the serum of TLR2-deficient mice significantly reduced TLR2/6 activity in *Bifidobacterium* and *Lactobacillus*, leading to microbial dysbiosis (van Bergenhenegouwen et al., 2014). Overall, intestinal EVs directly or indirectly interact with immune cells, IECs, and microbiota, thereby modulating immune and inflammation responses, intestinal barrier, and gut microbial structure.

The current studies on EV treatment of colitis lack distinct criteria. Herein, we conducted subgroup analyses on the animal species, colitis models, EV source, total doses of EVs, time of administration, delivery route, treatment frequency, and follow-up duration of these included studies. Although specific sources of heterogeneity remained unidentified, we observed that EVs performed better on most indicators in mouse experiments in TNBS-induced colitis models (except for IL-10) and in short-term follow-ups. The analysis revealed that different studies applied variable EV dosages. These subgroup analyses suggested that ≥200 mg may exert an optimal therapeutic effect (MPO activity is optimal at < 200 mg); however, there is an urgent need to compare the efficacy of different doses of EVs for colitis treatment. Notably, commercial kits were rather efficacious across all measures. In addition, the improvement of different indicators in the subgroups was variable with respect to the therapy timing, delivery methods, and treatment frequency. The optimal treatment plan and criteria for EV treatment need to be explored further.

The source of EVs in our analysis contains MSCs, microbiota, milk, plants, and macrophages.

MSCs have become promising candidates for the treatment of IBD due to their differentiation ability and anti-inflammatory properties (Wang et al., 2022). MSCs-EVs contribute to the self-renewal, immune regulation, expansion, and damage repair ability of stem cells (Li et al., 2020) and avoid the potential risks of stem cell transplantation compared to stem cell therapy; these molecules are easy to transport and store. Some studies have demonstrated the positive role of bone marrow mesenchymal stem cells (BMSCs) (Yang et al., 2015; Cao L et al., 2019; Yang R et al., 2020; Tolomeo et al., 2021; Zhu et al., 2022), human placental mesenchymal stem cells (hP-MSCs) (Duan et al., 2020), human umbilical cord-derived MSCs (hUC-MSCs) (Mao et al., 2017; Wu et al., 2018; Wang et al., 2020; Cai et al., 2021; Yang et al., 2021; Gan et al., 2022; Heidari et al., 2022; Liang et al., 2023), adipose-derived stem cells (ASCs) (Heidari et al., 2021; Qian et al., 2023), and olfactory ecto-mesenchymal stem cells (OE-MSCs) (Mao et al., 2017) in colitis, mainly through decreased pro-inflammatory cytokine levels, increased anti-inflammatory cytokine levels, and inhibition of the NF-κB signaling pathway. Interestingly, the therapeutic effect of MSCs-EVs is related to the maintenance of tight junctions (Duan et al., 2020; Liu L et al., 2021; Yang et al., 2021), regulation of ubiquitin modification levels (Wu et al., 2018; Wang et al., 2020), Treg/Th17 balance (Yang R et al., 2020; Yang et al., 2021; Heidari et al., 2022), inhibition of oxidative stress (Yang et al., 2015; Duan et al., 2020), apoptosis (Yang et al., 2015; Duan et al., 2020), and reduced NOD-like receptor thermal protein domain associated protein 3 (NLRP3) inflammasomes (Tong et al., 2021a; Tong et al., 2021b; Cai et al., 2021). The JAK/STAT (Cao L et al., 2019) and Wnt signaling pathways (Liang et al., 2023) have also been found to be involved in the effect of MSCs-EVs in colitis. Consistent with these findings, our study confirmed the effect of EVs on colitis, especially in HS.

Microbiota has long been under intensive focus in IBD research. Hence, microbiota transplantation (FMT) is considered an effective therapy for IBD given its ability to re-establish a healthy gut microbiome; however, its adverse events and the high risk to the IBD population should be noted (Dailey et al., 2019). Notably, microbial-derived EVs are key mediators of gut bacteria–host interactions and exhibit gentle growth and low risk. Some studies found that EVs derived from probiotics (*Lactobacillus paracasei* (Choi et al., 2020), *Lactobacillus rhamnosus* GG (Tong et al., 2021b), *Lactobacillus plantarum* Q7 (Hao et al., 2021)), commensal bacteria (*Clostridium butyricum* (Liang et al., 2022; Ma et al., 2022; Ma et al., 2023)), and parasites (*Trichinella spiralis* (Yang Y et al., 2020; Gao et al., 2021), *Giardia lamblia* (Kim et al., 2022), and *Nippostrongylus brasiliensis* (Eichenberger et al., 2018)) had therapeutic potential for colitis, effectuated by reducing the expression of the pro-inflammatory cytokines, elevating the expression of the anti-inflammatory cytokines and alleviating bacterial dysbiosis. The efficacy of microbiota-EVs on colitis (especially in MPO activity and IL-10) was consistent with the above findings. However, EVs originating from *Fusobacterium nucleatum* could promote gut barrier disruption in UC by activating RIPK1-mediated epithelial cell death (Liu L et al., 2021). *Fusobacterium nucleatum* produces multiple pathogenic factors and is enriched in the feces of IBD patients, thus deemed to be associated with the progression of the disease (Liu et al., 2020). Díaz-Garrido et al. (Díaz-Garrido et al., 2021) summarized microbiota-derived EVs in interkingdom communication in the gut and revealed their pivotal role in immunity, metabolism, and intestinal barrier integrity. These findings expand our knowledge of host-microbiota interactions and may help design novel and effective strategies for IBD and other gastrointestinal diseases as well as systemic diseases.

Previous studies have shown that abnormal immune responses contribute significantly to IBD pathogenesis. Immune-cell-derived EVs have attracted significant attention from researchers. Macrophage-derived EVs can rescue intestinal stem cells and improve enterocyte injury after radiation by regulating WNT function (Saha et al., 2016). The protective effects of M2 macrophage EVs on colitis are exerted through the CCL1/CCR8 axis (Yang et al., 2019) and LATS1/YAP/β-catenin axis (Deng et al., 2021). Although only one study addressed these aspects, the positive effect of macrophage-derived EVs on colitis was discovered. Moreover, other included studies confirmed that EVs promote macrophage polarization (Cao L et al., 2019; Tolomeo et al., 2021; Gao et al., 2022; Liang et al., 2022; Qian et al., 2023). IBD is characterized by neutrophil infiltration, which triggers the release of MPO into the extracellular space. Neutrophil-derived EVs exert anti-inflammatory functions (Soehnlein et al., 2017) via anti-inflammatory miRNAs (miR-23a, miR-155, miR-126, miR-150, and miR-451a) and induce anti-inflammatory macrophage polarization (Bui and Sumagin, 2019; Butin-Israeli et al., 2019; Youn et al., 2021). MPO damages the intestinal barrier by producing oxidative radicals; however, our analysis found that EVs from any source can suppress the MPO activity. Another study showed that EVs released by granulocytic myeloid-derived suppressor cells (G-MDSC) promote Tregs expansion and inhibit Th1 cell proliferation, which in turn attenuates DSS-induced colitis (Wang et al., 2016). The key role of DC-derived EVs in regulating inflammation in IBD is also shown by inhibiting T-cell proliferation (Kim et al., 2007; Tkach et al., 2017).

In addition to host cells and the microenvironment, milk and plants are also significant and safe sources of EVs (Di Gioia, Hossain, and Conese, 2020; Ocansey et al., 2020). Numerous studies have indicated that EVs from any source of milk (human or different animal) improve colitis by promoting the proliferation and viability of IECs, increasing stem cell activity, shaping the intestinal microbiome, ameliorating inflammation, and enhancing intestinal immunity (Chen et al., 2016; Hock et al., 2017; Martin et al., 2018; Benmoussa et al., 2019; Gao et al., 2019; Reif et al., 2020; Tong et al., 2020; Tong et al., 2021a; Du et al., 2022; Han et al., 2022; Tong et al., 2023). As expected, we observed various improvements in colitis-related indicators by milk-derived EVs. Several studies have reported plant-derived EVs’ potential anti-inflammatory and anti-cancer properties (Wang et al., 2015; Zhang et al., 2016; Deng et al., 2017; Teng et al., 2018; Cao M et al., 2019; Liu B et al., 2021; Sundaram et al., 2022). Gao et al. (2022)) demonstrated the excellent anti-inflammatory efficacy of turmeric-derived EVs as they restore intestinal barrier integrity, regulate the macrophage phenotype, and reshape the gut microbiota. EVs derived from another medical herb, *Curcuma longa*, may inactivate NF-κB to alleviate colitis (Zhang et al., 2019). Similar results were observed in EV-like nanoparticles (ELN) from grapes (Ju et al., 2013) and broccoli (Deng et al., 2017). Our results reconfirmed the therapeutic effects of milk- and plant-derived EVs, although the improvement degree varies among the colitis-related indicators.

Although human studies of EV therapy for IBD are limited, existing evidence shows the potential of EVs to relieve colon inflammation. After injection of EVs extracted from MSCs in five patients with refractory perianal CD fistulas, four patients responded to therapy, among whom three exhibited complete healing while one did not report any improvement or active discharge from the fistula site (Nazari et al., 2022). Nevertheless, neither systemic nor local adverse effects were reported in the 5 patients (Nazari et al., 2022).

The promising role of EVs as drug carriers for IBD should also be examined. Conventional drug therapy for IBD relies on high-dose administration for a prolonged period, after which toxicity accumulation can lead to serious adverse reactions. EV-based drug delivery systems, with several advantages (low immunogenicity, high biocompatibility, and stability), open new avenues for delivering therapeutic agents to target cells in intestinal inflammatory sites, enhancing permeability and retention, reducing the side effects and toxicity of the drugs, and improving therapeutic efficiency (Rezaie et al., 2022). However, the application of EVs as drug carriers is posed with several challenges and needs to be explored further.

Supposedly, the role of EVs in IBD is as a diagnostic marker with the advantage of stable content (protected by the EV membrane) as opposed to free circulating RNAs or proteins (Ocansey et al., 2020; Valter et al., 2021; Li et al., 2022; Tian et al., 2023). A potential EV biomarker for IBD is Annexin-1; it shows higher levels in serum EVs isolated from IBD patients with active mucosal inflammation than in healthy controls (HCs) and was positively correlated with the severity of inflammation (Leoni et al., 2015). Another study compared the protein profiles of EVs isolated from the saliva of UC and CD patients and HCs and identified eight proteins: PSMA7, BASP1, TKT, TLN1, WDR1, NUCB2, PSMB7, and IGHV4OR. Since these proteins were only detected in the EVs of IBD patients, they were potential biomarkers for IBD (Zheng et al., 2017). Chen et al. (Chen et al., 2022) observed 3.33-fold increased serum EVs miR-144-3p levels in CD patients compared to the HCs. The study also showed that the EVs miR-144-3p levels are positively associated with the endoscopic score of CD and Rutgeerts score. Thus, the serum EVs miR-144-3p, a putative biomarker of mucosal inflammation and penetrating CD, may be more accurate in identifying endoscopic CD recurrence after intestinal resection than C-reactive protein (CRP) detection.

Taken together, this study supports the use of EVs as a novel therapy for IBD. The meta-analysis results provide a valuable reference for future preclinical and clinical studies of IBD with significant implications for human health.

## 5 Strengths and limitations

Several studies have focused on animal models of colitis, while human RCTs are lacking. To the best of our knowledge, this is the first systematic review and meta-analysis to comprehensively summarize the efficiency of EVs in colitis therapy among animal trials. Nevertheless, the limitations cannot be ignored. First, we include studies published in only English. Studies with complete data were not available, and unpublished data might influence our conclusions. Second, although varying degrees of heterogeneity were observed among studies, further subgroup and sensitivity analyses did not identify the source. We also observed differences in detail in studies related to EV treatment of colitis (including sources, dose, and frequency) that would be considered carefully in future studies. Detailed criteria for EV treatment of colitis also need to be established. Third, publication bias was observed in the included studies that should be taken into account while interpreting the results. Furthermore, although no adverse events were reported, none of the included studies conducted formal tests to investigate the safety of EVs. Therefore, appropriately designed large animal and preclinical studies are imperative.

## 6 Conclusion

Our meta-analysis results supported the protective effect of Evs on colitis rodent models based on their potential role in IBD therapy, propelling the field toward clinical studies.

## Data Availability

The original contributions presented in the study are included in the article/Supplementary Material, further inquiries can be directed to the corresponding authors.
