# Supplementary material for: Efficacy of extracellular vesicles as a cell-free therapy in colitis: a systematic review and meta-analysis of animal studies
Source: Front Pharmacol. 2023 Oct 26;14:1260134. doi: 10.3389/fphar.2023.1260134 (PMC10637393; doi:10.3389/fphar.2023.1260134)
Supplement: Supplementary file 1 [file DataSheet3.doc]

**Supplementary material 3**

**Fig. S1a. Subgroup analysis by animals species for DAI.**

**
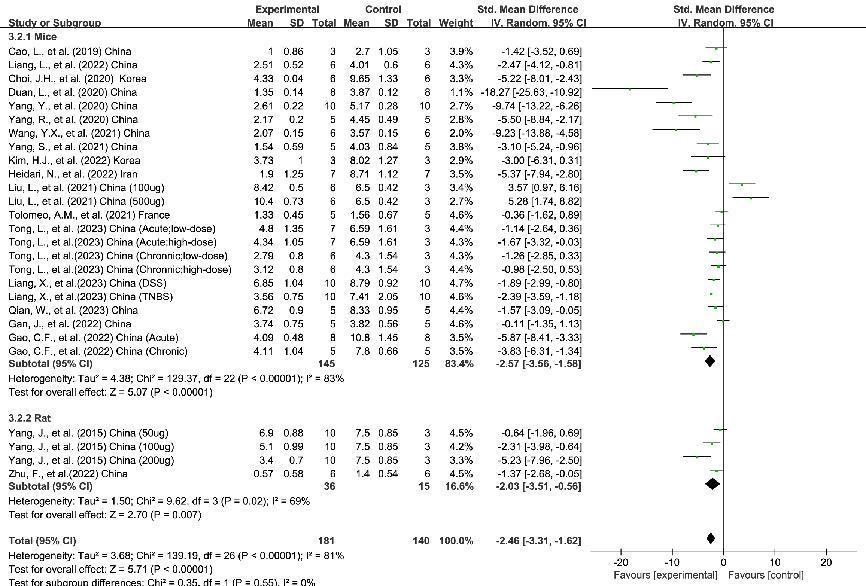
**

**Fig. S1b. Subgroup analysis by model for the DAI.**

**
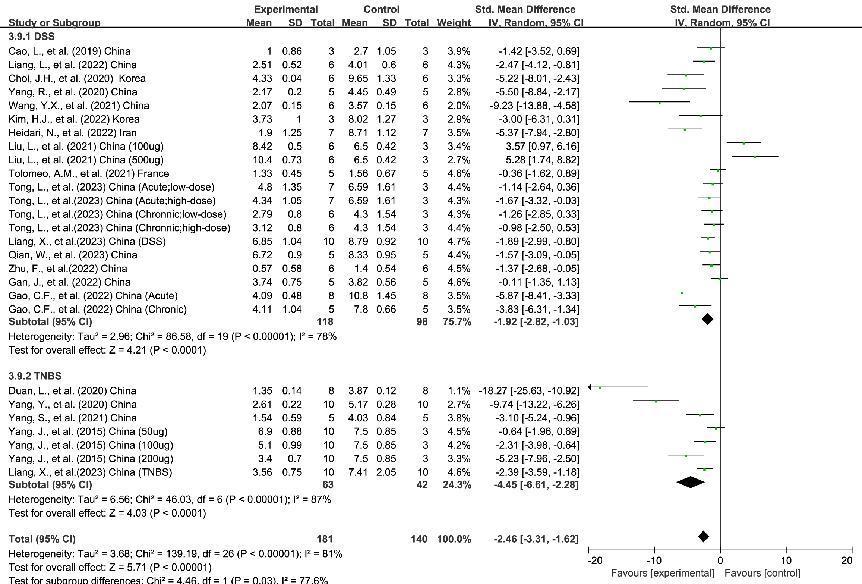
**

**Fig. S1c. Subgroup analysis by the source of EVs for DAI.**

**
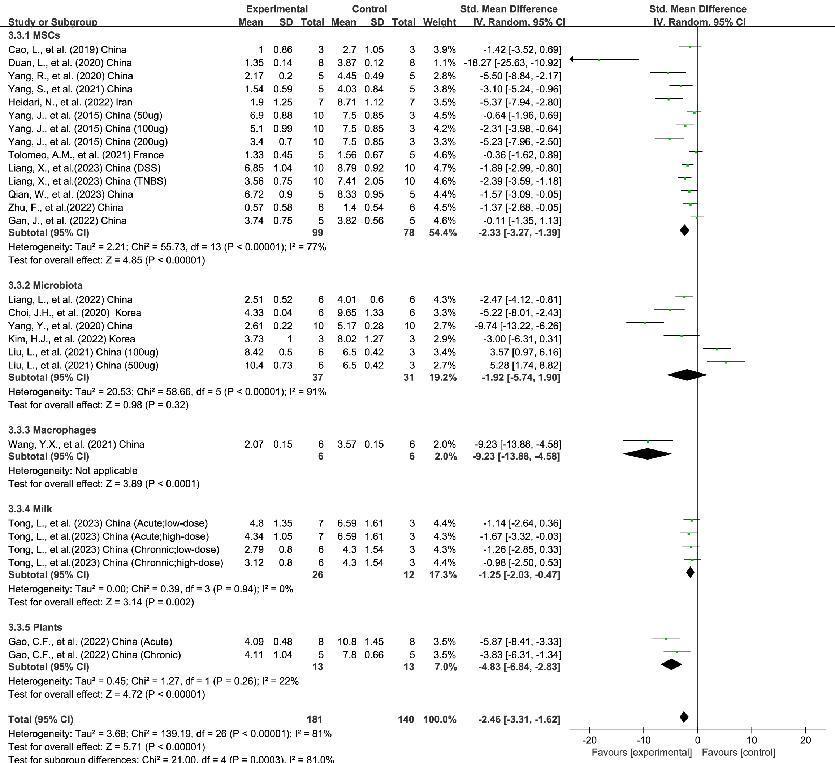
**

**Fig. S1d. Subgroup analysis by the therapy dose for DAI.**

**
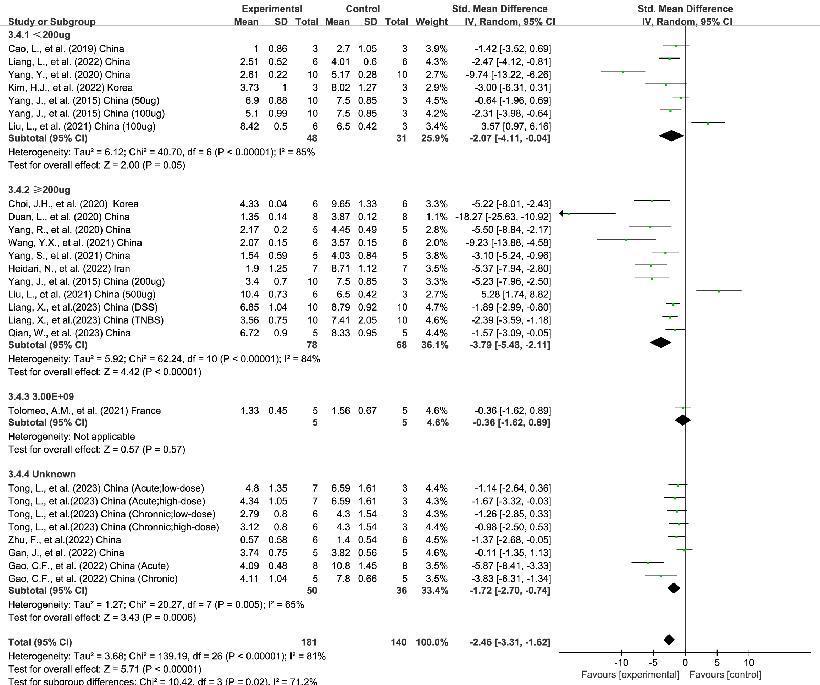
**

**Fig. S1e. Subgroup analysis by the isolation method of EVs for DAI.**

**
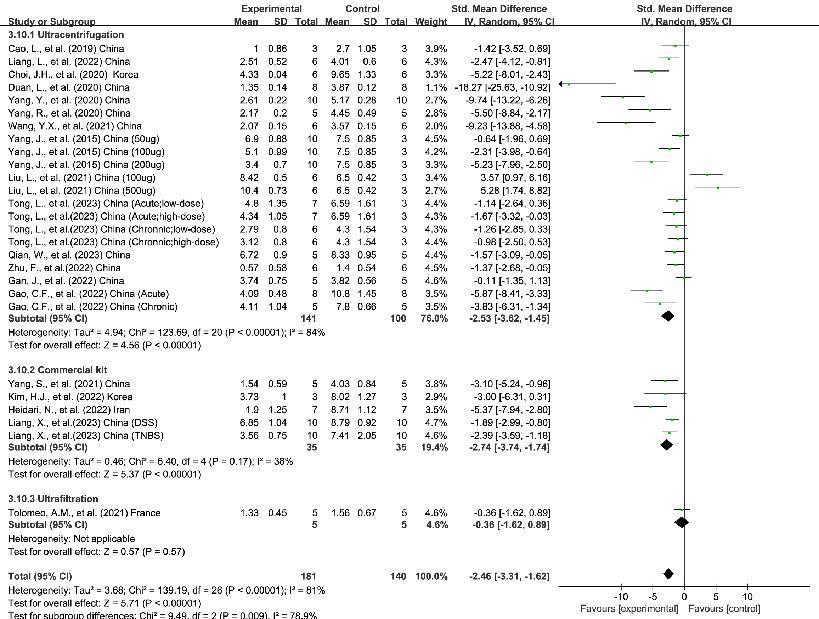
**

**Fig. S1f. Subgroup analysis by the delivery route for DAI.**

**
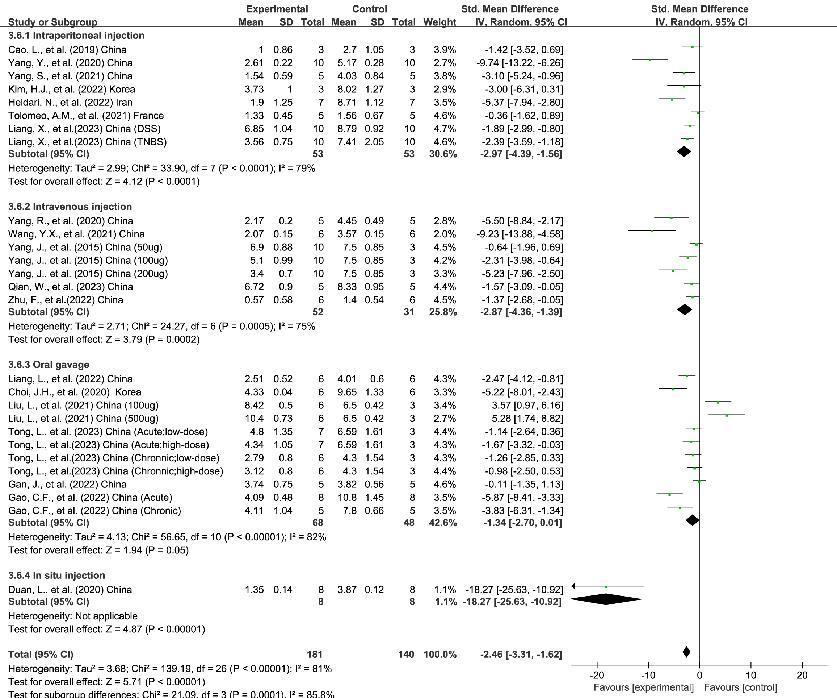
**

**Fig. S1g. Subgroup analysis by the therapy time for DAI.**

**
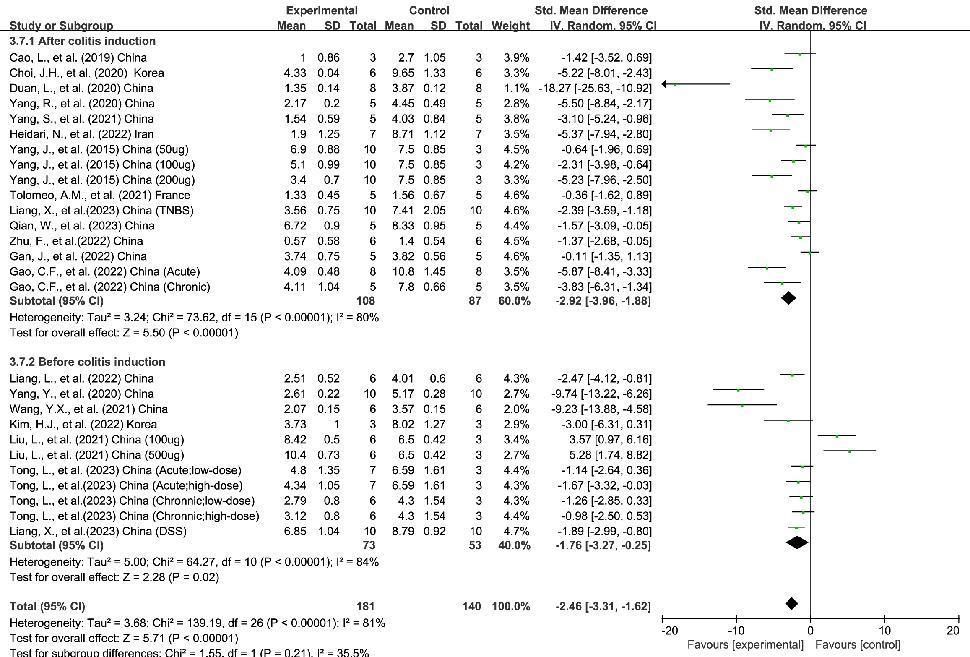
**

**Fig. S1h. Subgroup analysis by the treatment frequency for DAI.**


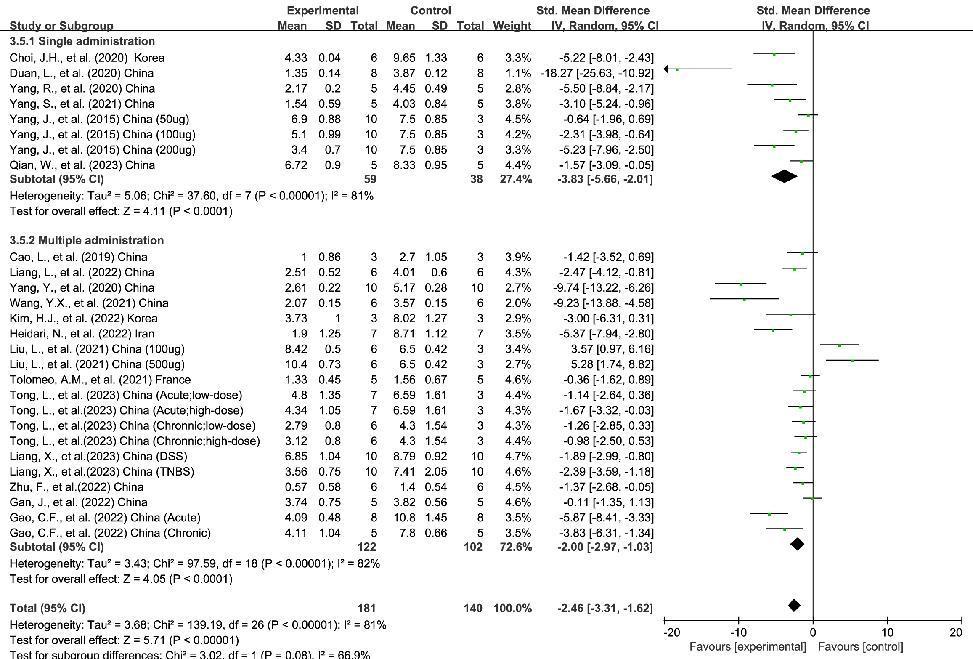


**Fig. S1i. Subgroup analysis by the follow-up duration for DAI.**

**
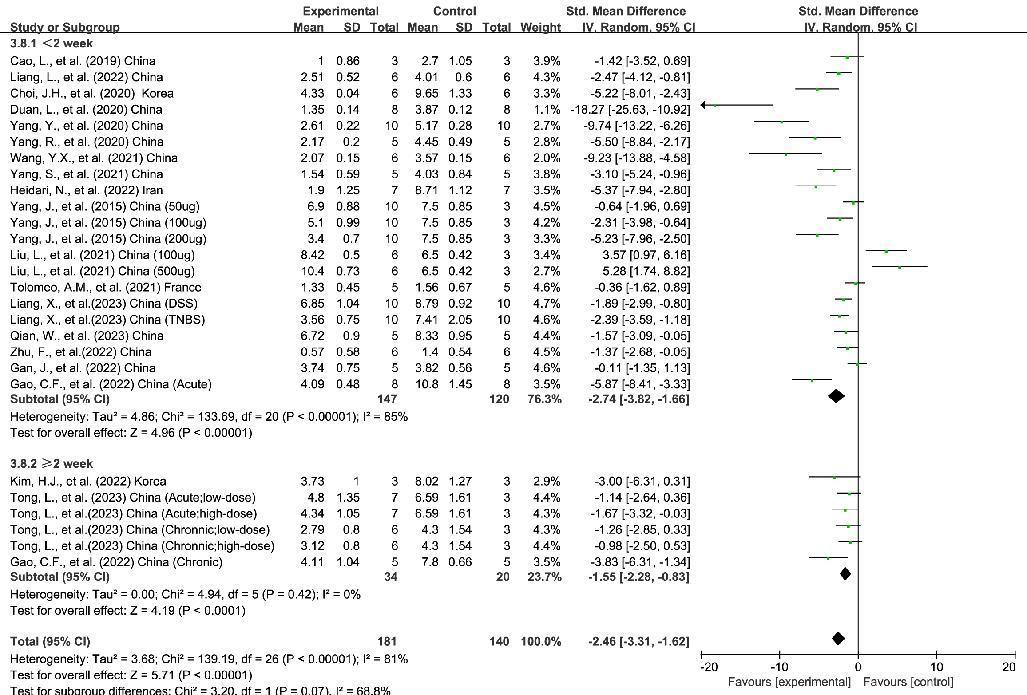
**

**Fig. S2a. Subgroup analysis by animals species for MPO activity.**

**
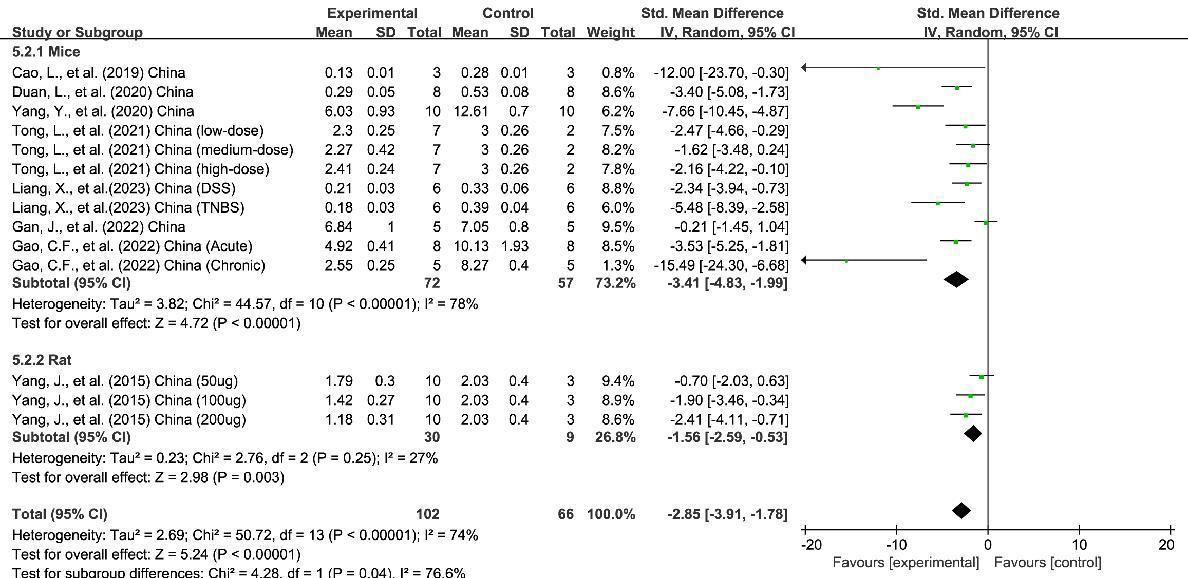
**

**Fig. S2b. Subgroup analysis by model for MPO activity.**

**
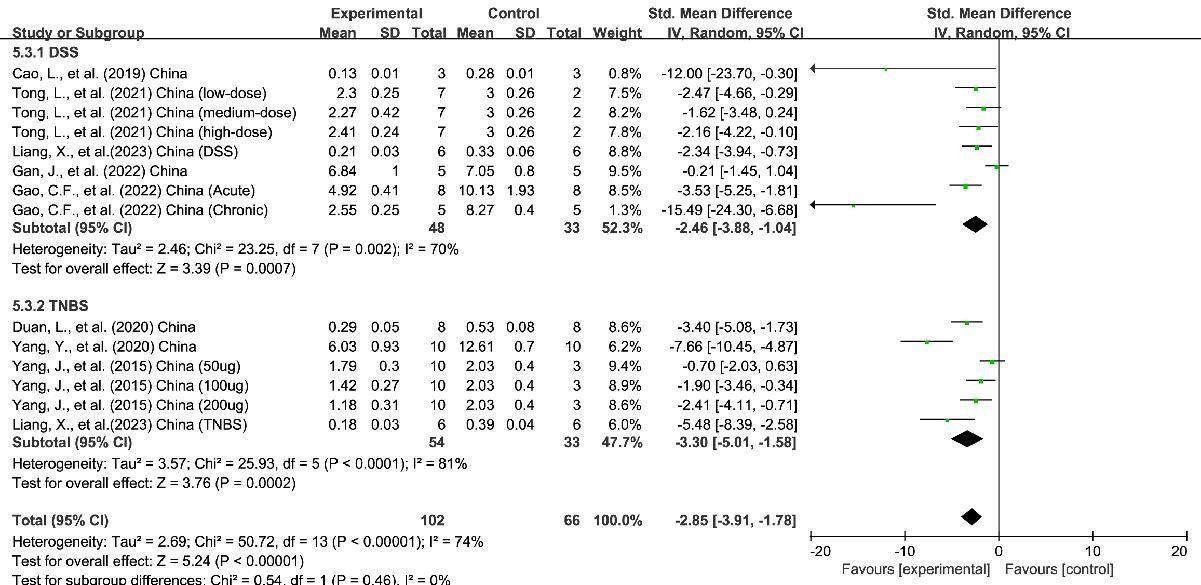
**

**Fig. S2c. Subgroup analysis by the source of EVs for MPO activity.**

**
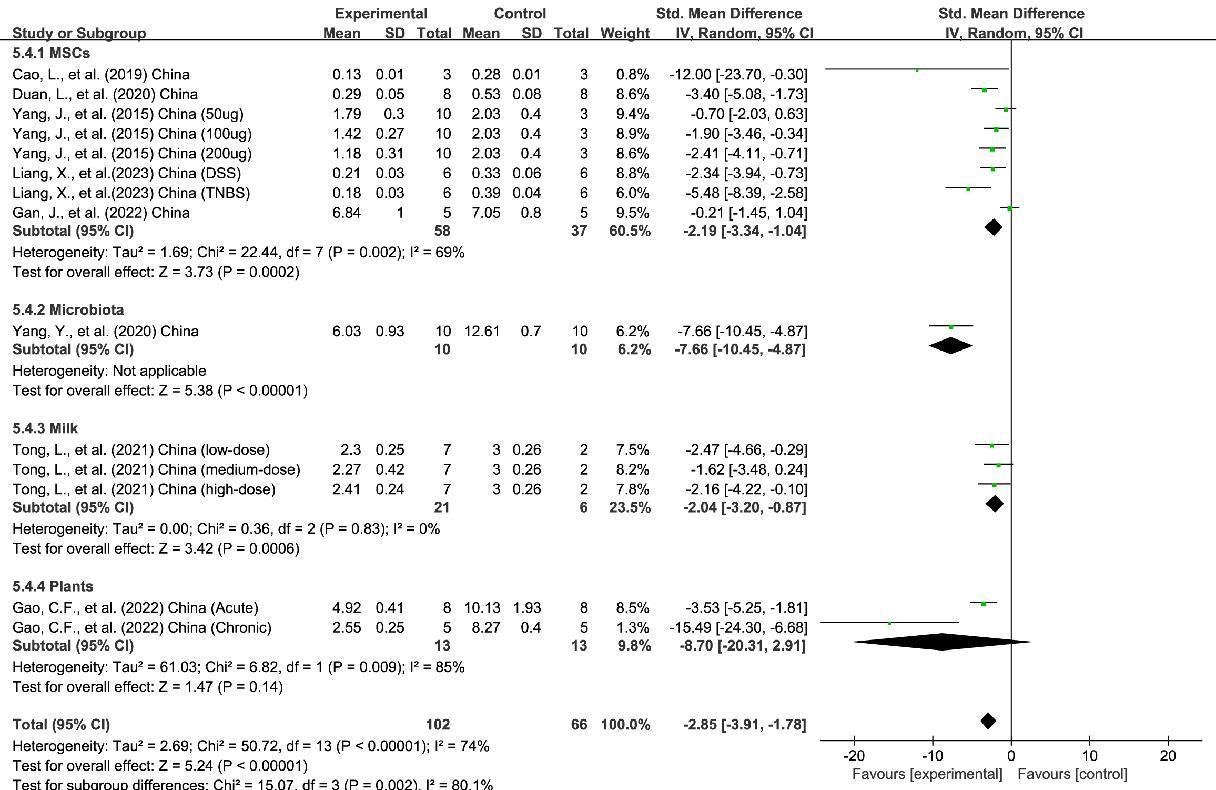
**

**Fig. S2d. Subgroup analysis by the therapy dose for MPO activity.**

**
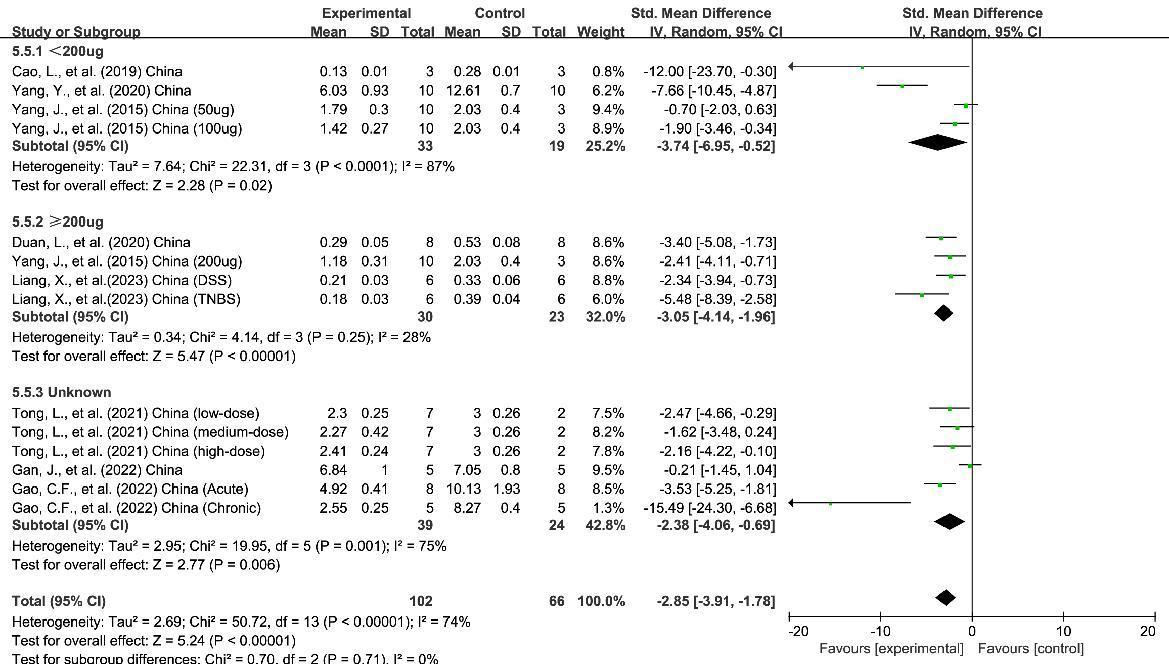
**

**Fig. S2e. Subgroup analysis by the isolation method of EVs for MPO activity.**

**
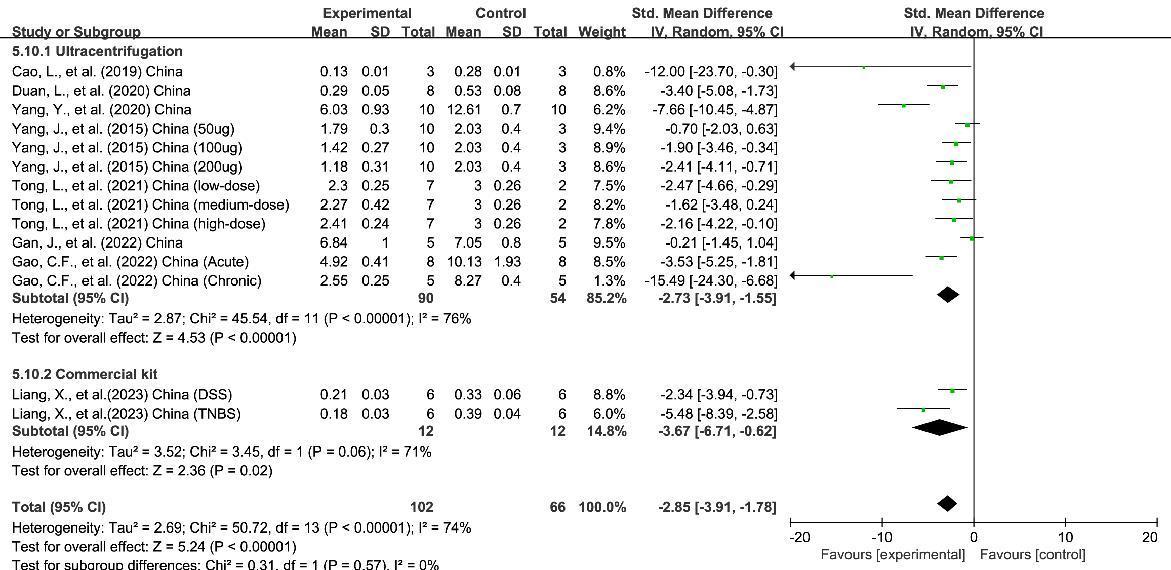
**

**Fig. S2f. Subgroup analysis by the delivery route for MPO activity.**

**
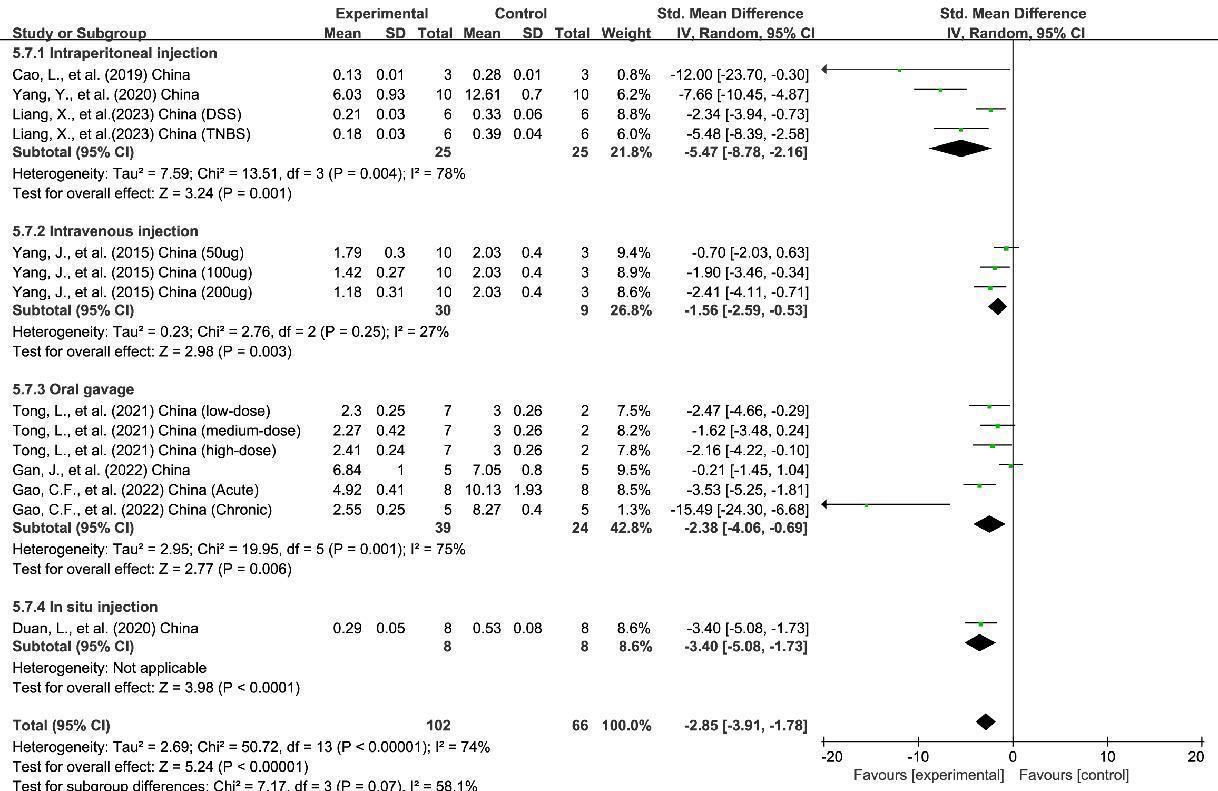
**

**Fig. S2g. Subgroup analysis by the therapy time for MPO activity.**

**
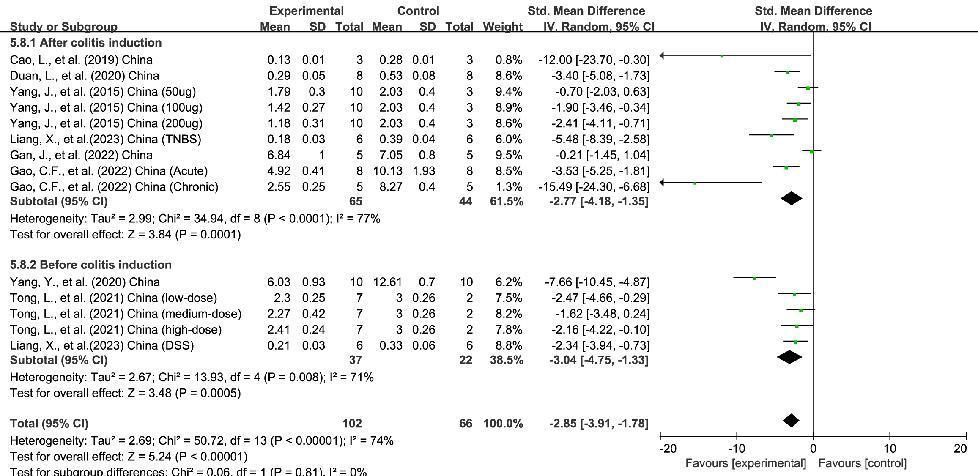
**

**Fig. S2h. Subgroup analysis by the treatment frequency for MPO activity.**

**
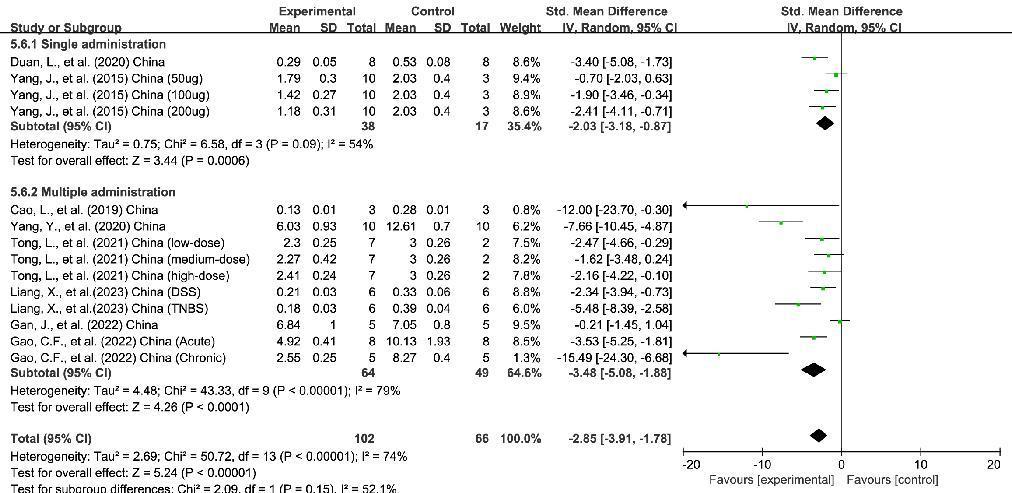
**

**Fig. S2i. Subgroup analysis by the follow-up duration for MPO activity.**

**
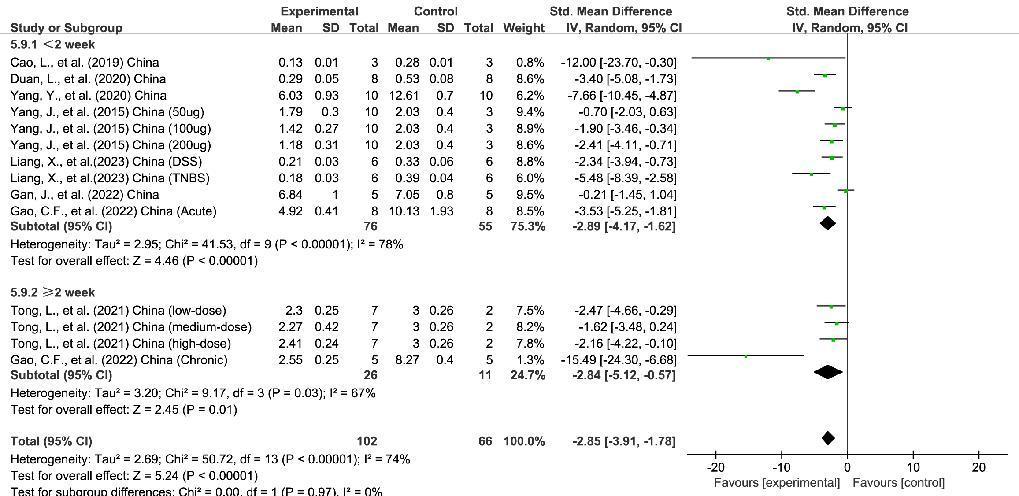
**

**Fig. S3a. Subgroup analysis by model for HS.**

**
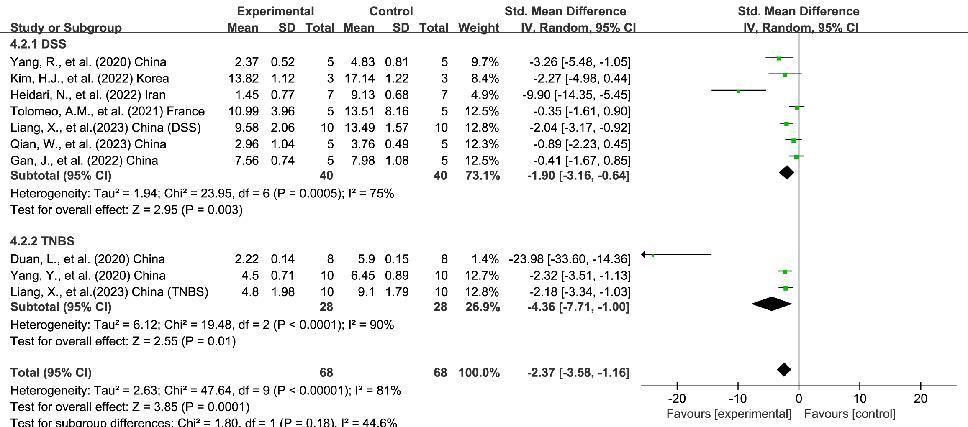
**

**Fig. S3b. Subgroup analysis by the source of EVs for HS.**

**
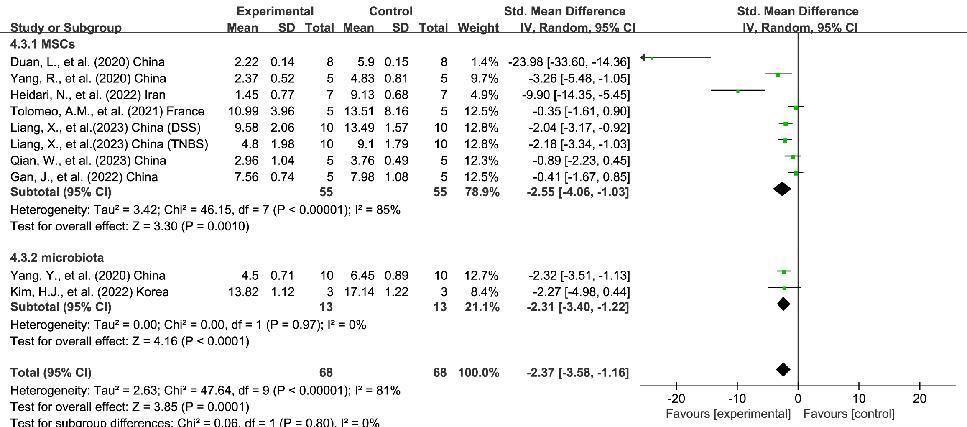
**

**Fig. S3c. Subgroup analysis by the therapy dose for HS.**

**
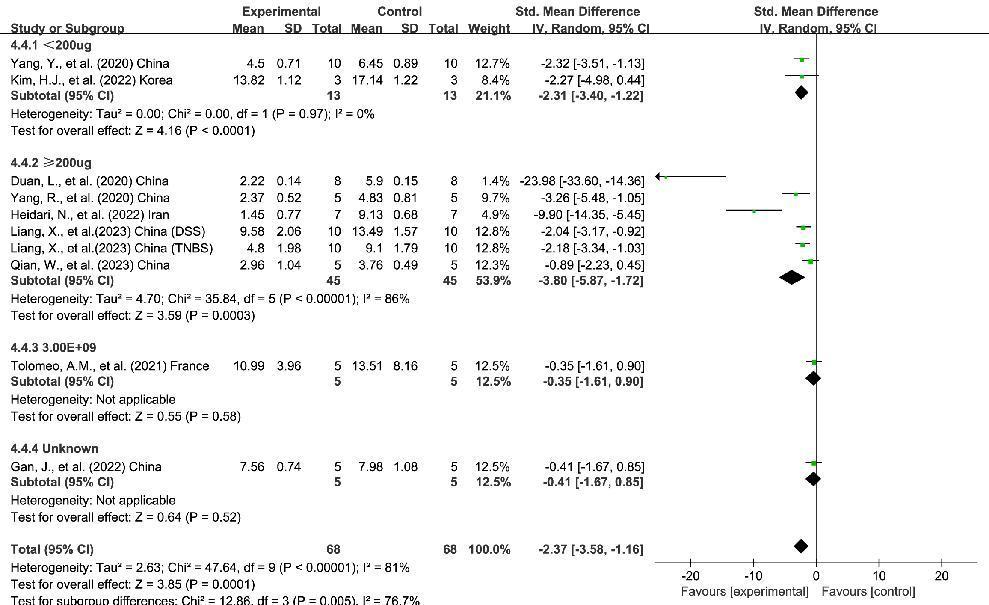
**

**Fig. S3d. Subgroup analysis by the isolation method of EVs for HS.**

**
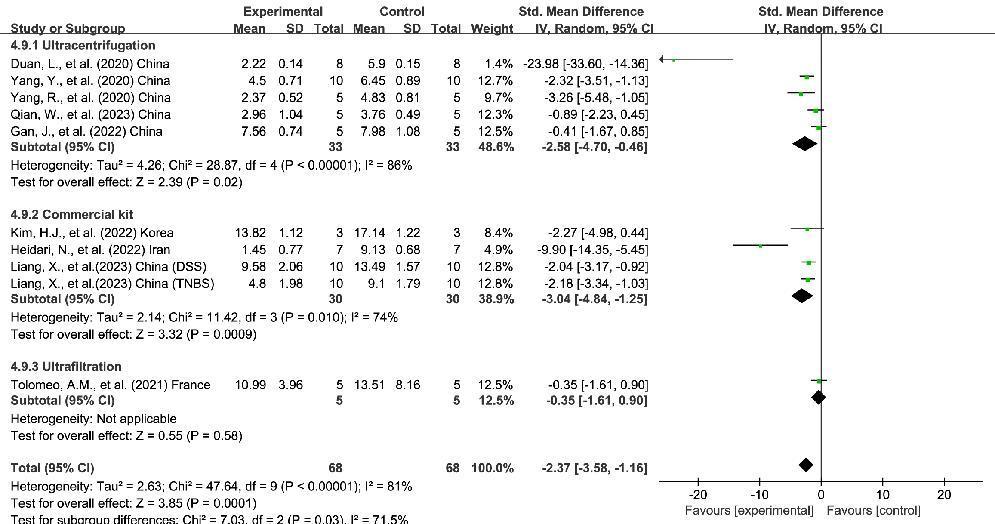
**

**Fig. S3e. Subgroup analysis by the delivery route for HS.**

**
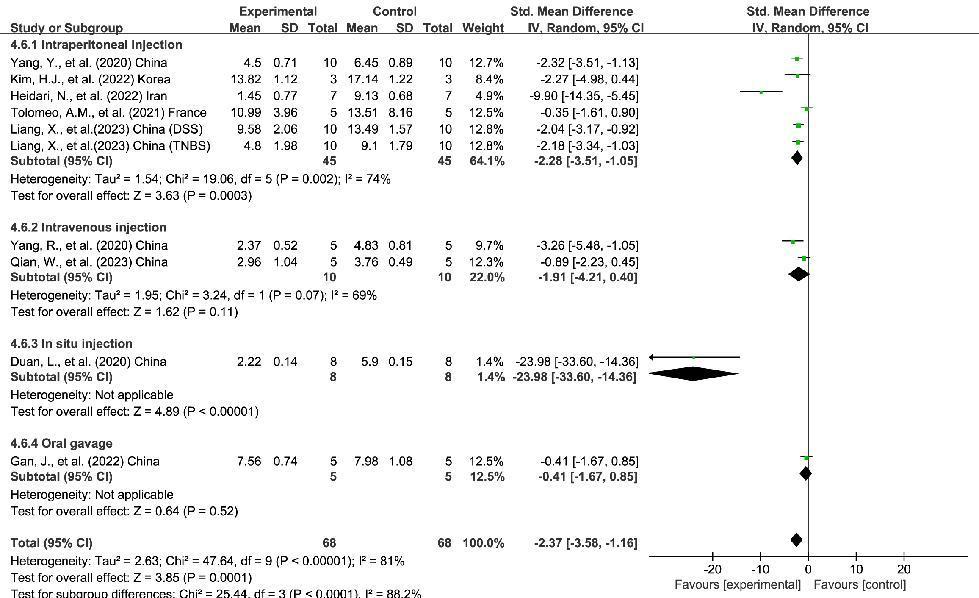
**

**Fig. S3f. Subgroup analysis by the therapy time for HS.**

**
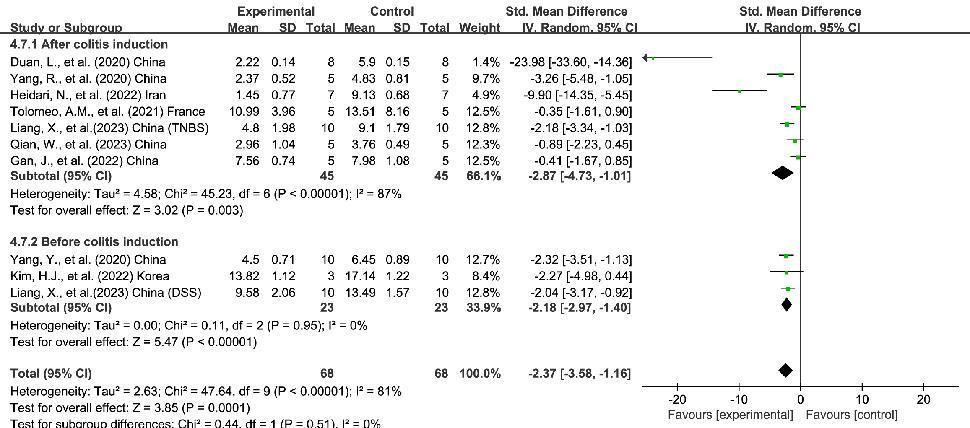
**

**Fig. S3g. Subgroup analysis by the treatment frequency for HS.**

**
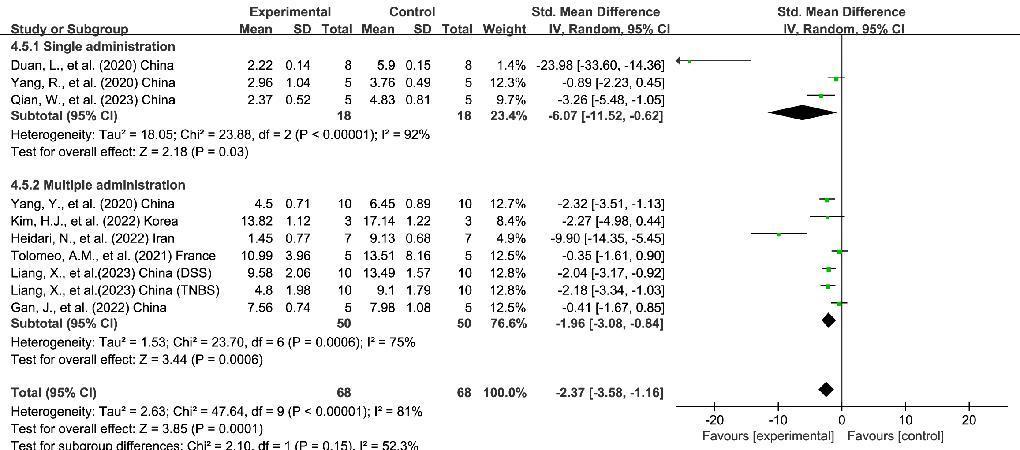
**

**Fig. S3h. Subgroup analysis by the follow-up duration for HS.**

**
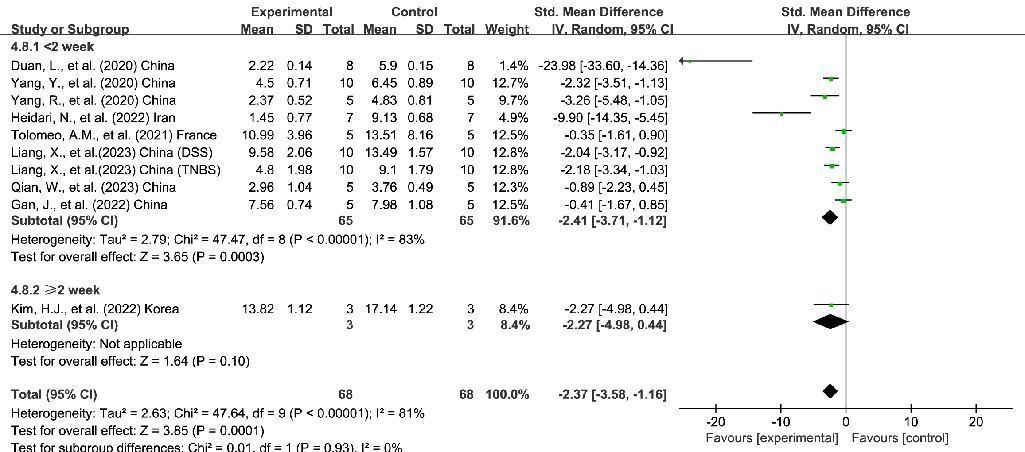
**

**Fig. S4a. Subgroup analysis by animals species for IL-1β.**

**
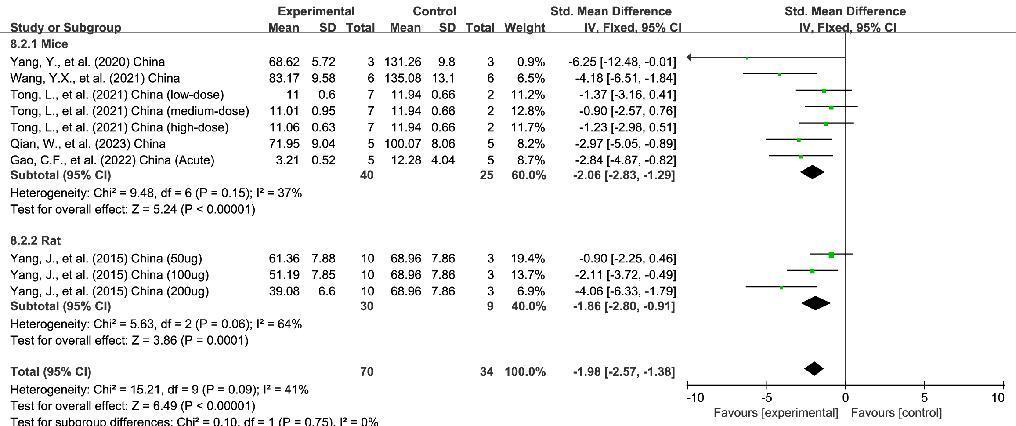
**

**Fig. S4b. Subgroup analysis by model for IL-1β.**

**
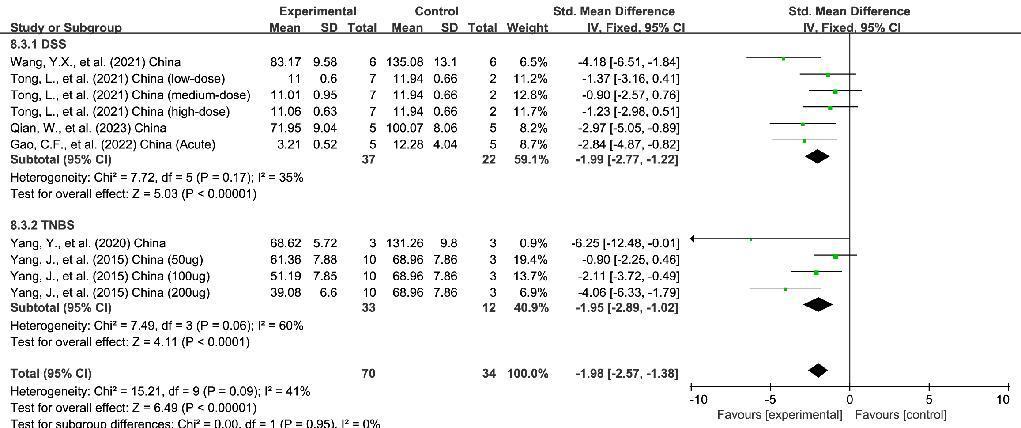
**

**Fig. S4c. Subgroup analysis by the source of EVs for IL-1β.**

**
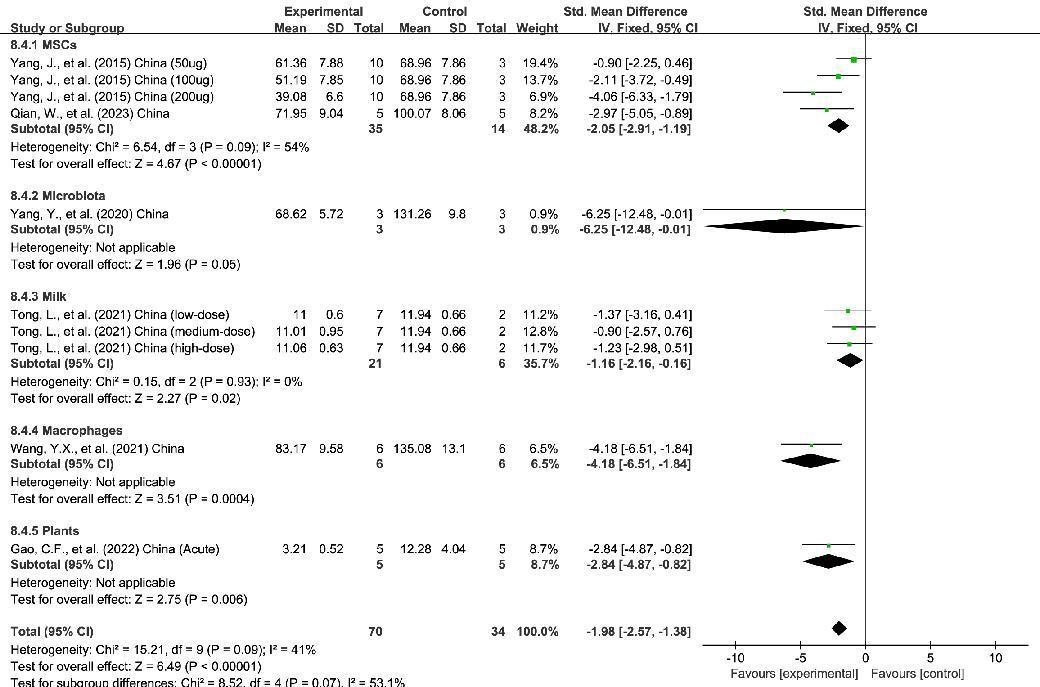
**

**Fig. S4d. Subgroup analysis by the therapy dose for IL-1β.**

**
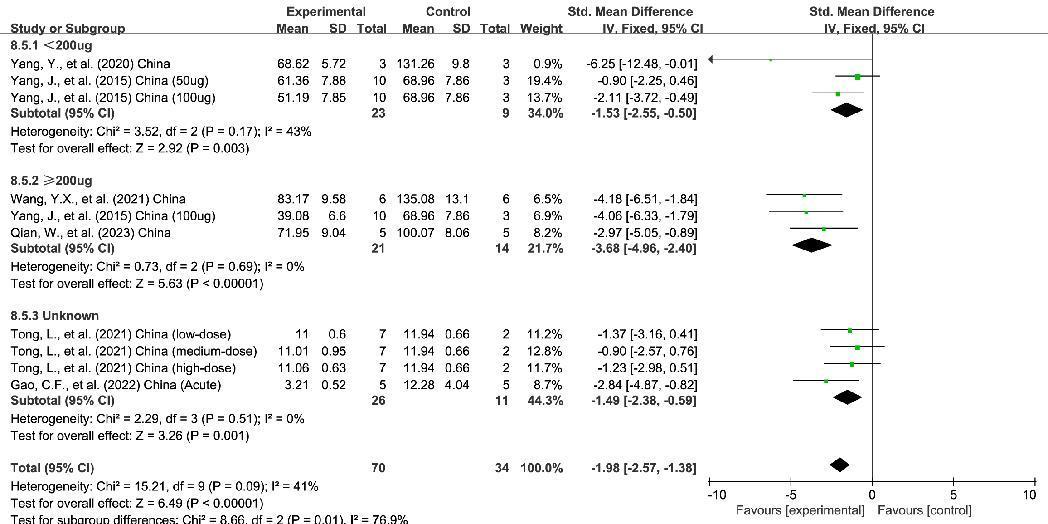
**

**Fig. S4e. Subgroup analysis by the delivery route for IL-1β.**

**
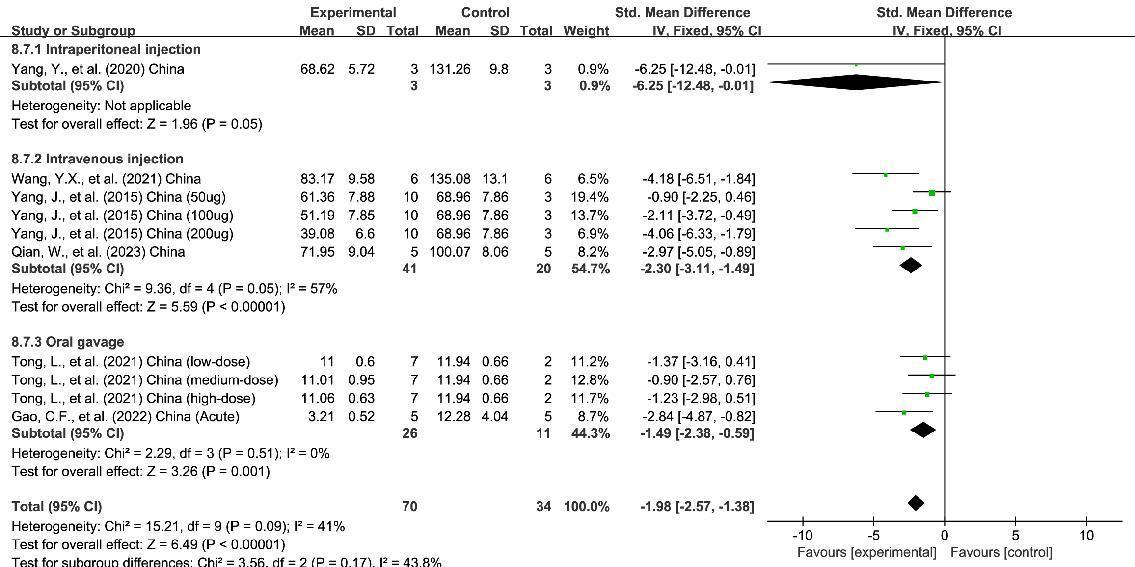
**

**Fig. S4f. Subgroup analysis by the therapy time for IL-1β.**

**
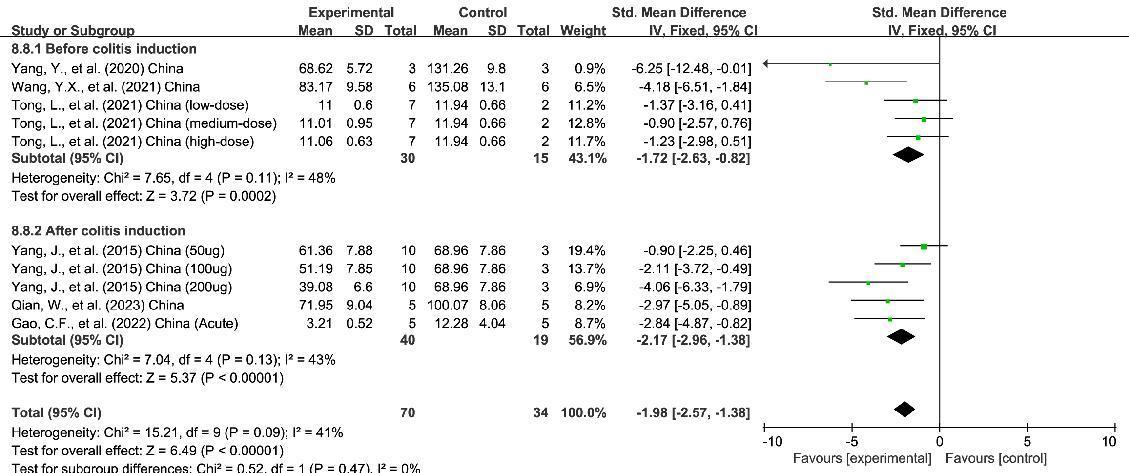
**

**Fig. S4g. Subgroup analysis by the treatment frequency for IL-1β.**

**
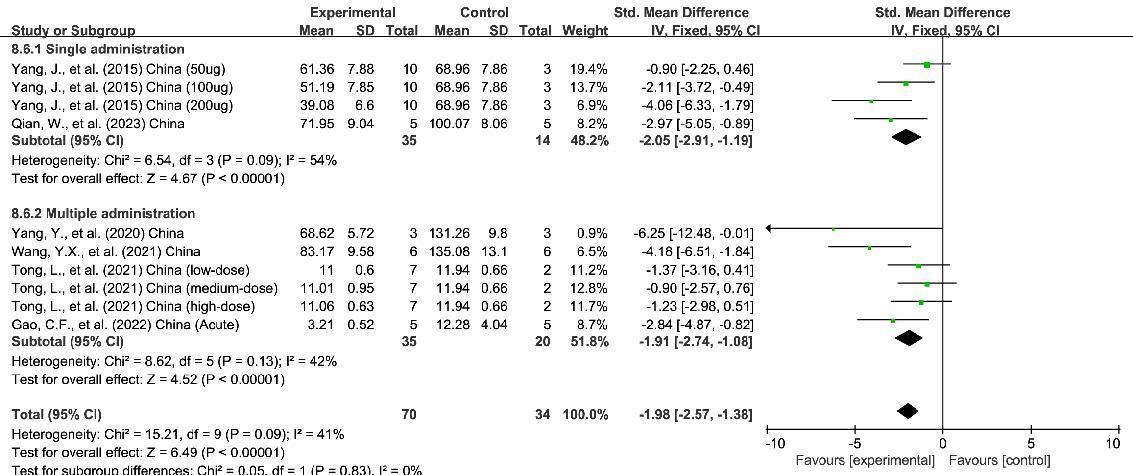
**

**Fig. S4h. Subgroup analysis by the follow-up duration for IL-1β.**

**
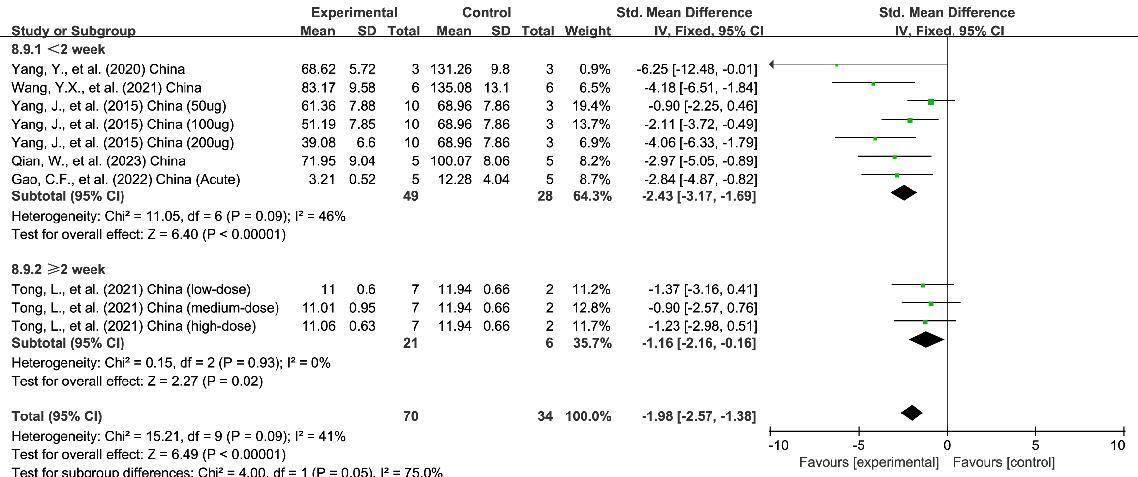
**

**Fig. S5a. Subgroup analysis by animals species for IL-10.**

**
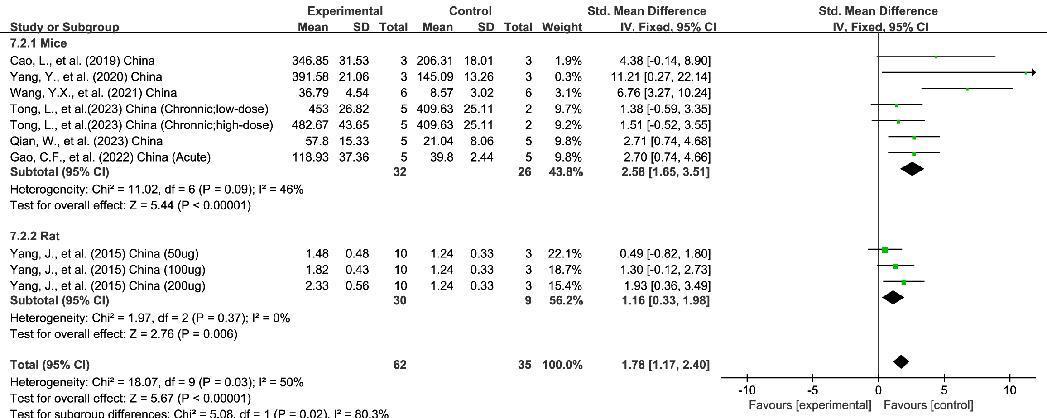
**

**Fig. S5b. Subgroup analysis by model for IL-10.**

**
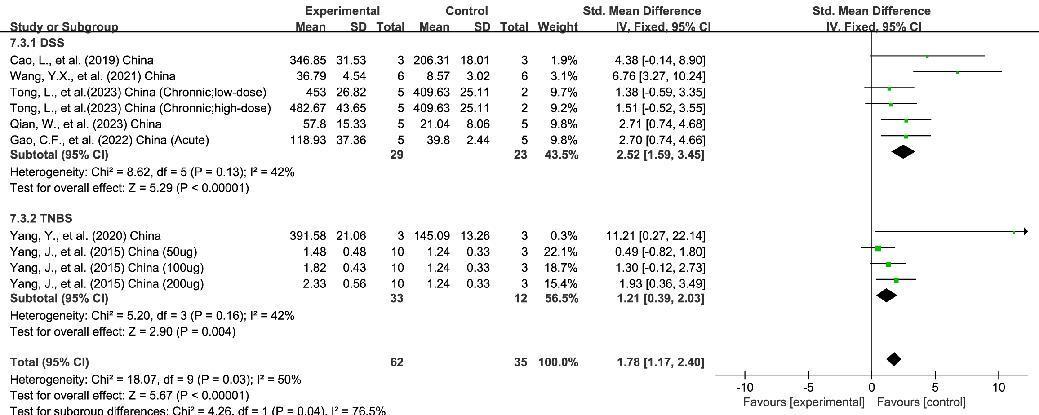
**

**Fig. S5c. Subgroup analysis by the source of EVs for IL-10.**

**
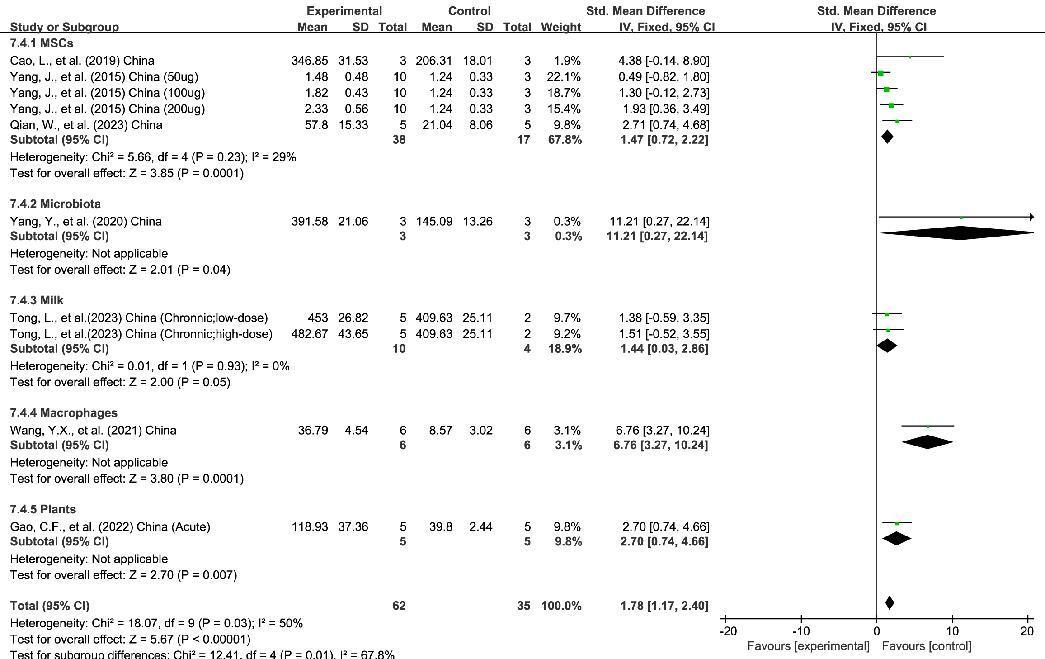
**

**Fig. S5d. Subgroup analysis by the therapy dose for IL-10.**

**
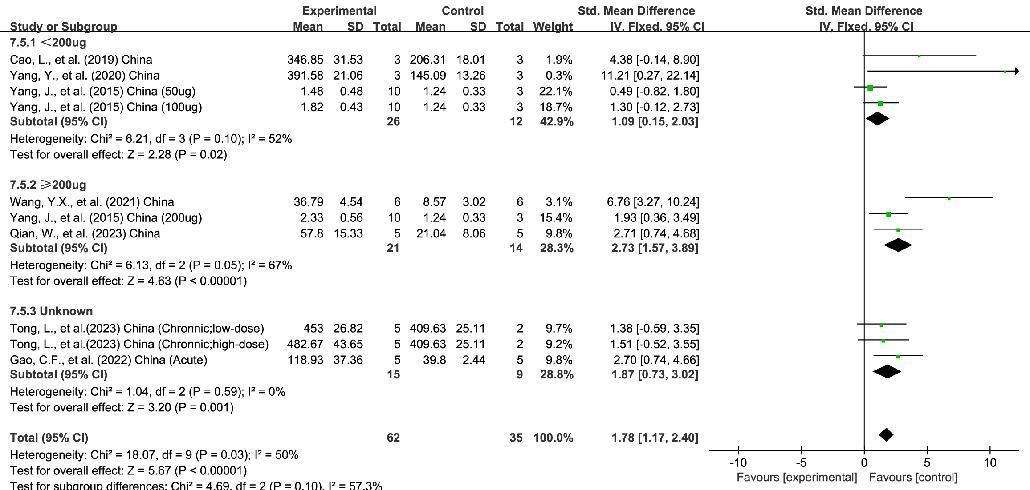
**

**Fig. S5e. Subgroup analysis by the delivery route for IL-10.**

**
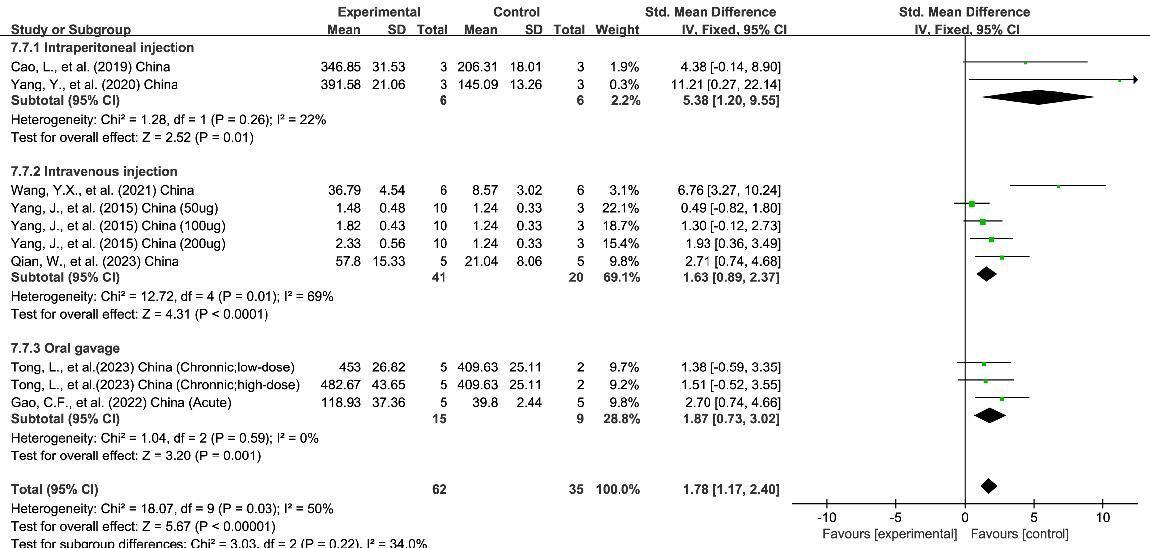
**

**Fig. S5f. Subgroup analysis by the therapy time for IL-10.**

**
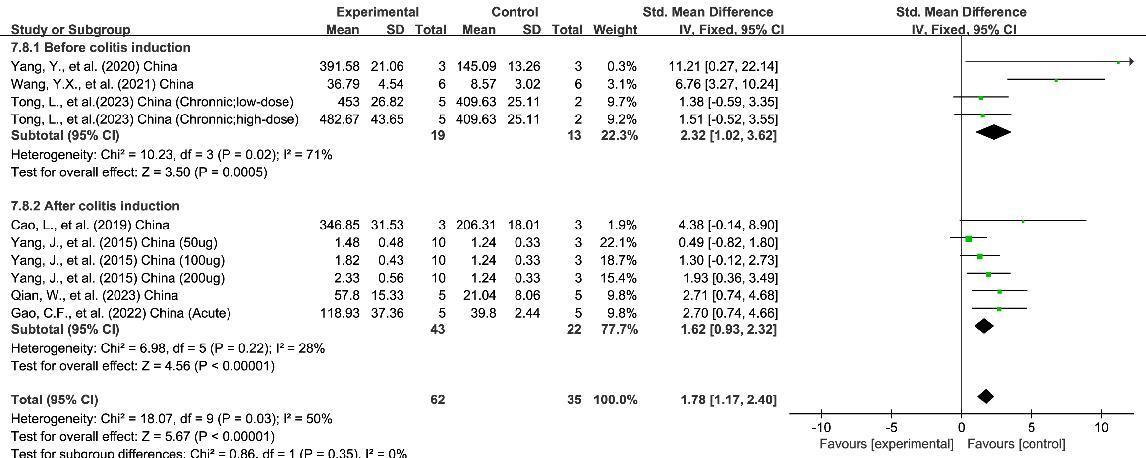
**

**Fig. S5g. Subgroup analysis by the treatment frequency for MPO activity.**

**
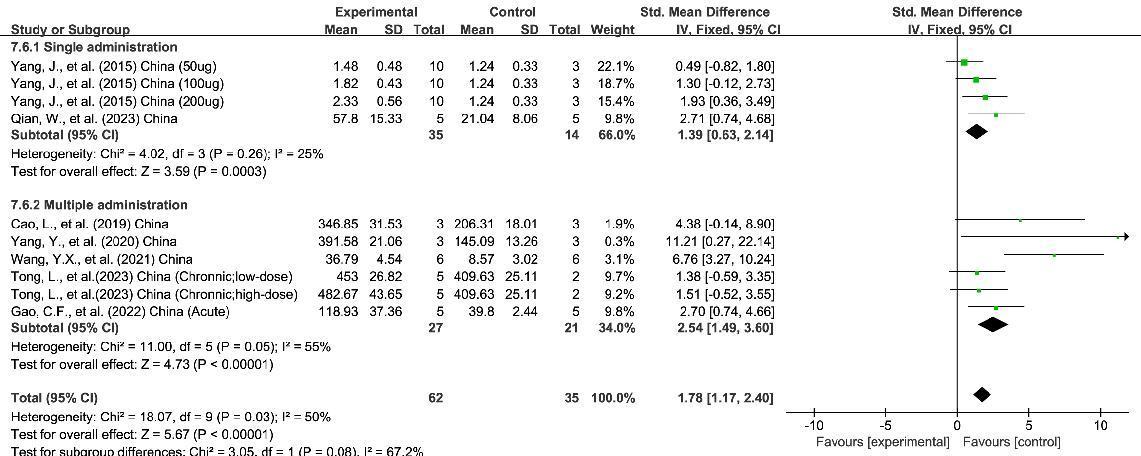
**

**Fig. S5h. Subgroup analysis by the follow-up duration for MPO activity.**

**
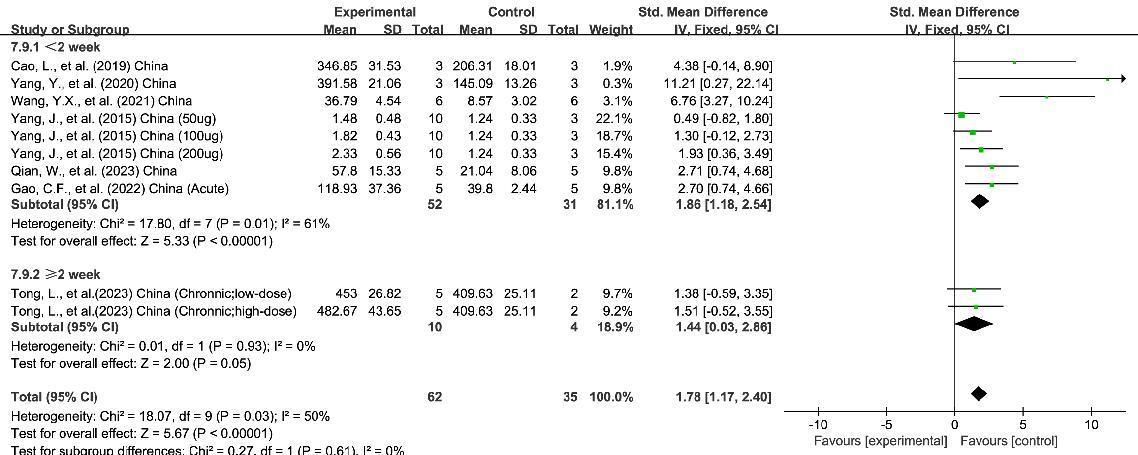
**
