# Supplementary material for: Efficacy of extracellular vesicles as a cell-free therapy in colitis: a systematic review and meta-analysis of animal studies
Source: Front Pharmacol. 2023 Oct 26;14:1260134. doi: 10.3389/fphar.2023.1260134 (PMC10637393; doi:10.3389/fphar.2023.1260134)
Supplement: Supplementary file 2 [file DataSheet2.docx]

**Table S1 Subgroup analysis of the DAI**

| **Analysis** | **Trials (n)** | **Analyzed (n)** | **SMD (95% CI)** | **p-value** | **I^2^** |
| --- | --- | --- | --- | --- | --- |
| Overall | 27 | 321 | -2.46 [-3.31, -1.62] | <0.05 | 81% |
| **Animal species** |  |  |  | **0.55** |  |
| Mice | 23 | 270 | -2.57 [-3.56, -1.58] | <0.05 | 83% |
| Rat | 4 | 51 | -2.03 [-3.51, -0.56] | <0.05 | 69% |
| **Models** |  |  |  | **<0.05** |  |
| DSS | 20 | 216 | -1.92 [-2.82, -1.03] | <0.05 | 78% |
| TNBS | 7 | 105 | -4.45 [-6.61, -2.28] | <0.05 | 87% |
| **EVs source** |  |  |  | **<0.05** |  |
| MSCs | 14 | 177 | -2.33 [-3.27, -1.39] | <0.05 | 77% |
| Microbiota | 6 | 68 | -1.92 [-5.74, 1.90] | 0.32 | 91% |
| Milk | 4 | 38 | -1.25 [-2.03, -0.47] | <0.05 | 0% |
| Plants | 2 | 26 | -4.83 [-6.84, -2.83] | <0.05 | 22% |
| Macrophages | 1 | 12 | -9.23 [-13.88, -4.58] | <0.05 | ---- |
| **Total doses of EVs** |  |  |  | **<0.05** |  |
| ＜200ug | 7 | 79 | -2.07 [-4.11, -0.04] | 0.05 | 85% |
| ≥200ug | 11 | 146 | -3.79 [-5.48, -2.11] | <0.05 | 84% |
| 3.00E+09 | 1 | 10 | -0.36 [-1.62, 0.89] | 0.57 | ---- |
| Unknown | 8 | 86 | -1.72 [-2.70, -0.74] | <0.05 | 65% |
| **Time of administration** |  |  |  | **0.21** |  |
| Before colitis induction | 11 | 126 | -1.76 [-3.27, -0.25] | <0.05 | 84% |
| After colitis induction | 16 | 195 | -2.92 [-3.96, -1.88] | <0.05 | 80% |
| **Delivery route** |  |  |  | **<0.05** |  |
| intraperitoneal injection | 8 | 106 | -2.97 [-4.39, -1.56] | <0.05 | 79% |
| intravenous injection | 7 | 83 | -2.87 [-4.36, -1.39] | <0.05 | 75% |
| oral gavage | 11 | 116 | -1.34 [-2.70, 0.01] | 0.05 | 82% |
| *in situ* injection | 1 | 16 | -18.27 [-25.63, -10.92] | <0.05 | ---- |
| **Treatment** **frequency** |  |  |  | **0.08** |  |
| Single administration | 8 | 97 | -3.83 [-5.66, -2.01] | <0.05 | 81% |
| Multiple administration | 19 | 224 | -2.00 [-2.97, -1.03] | <0.05 | 82% |
| **Follow-up duration** |  |  |  | **0.07** |  |
| <2 week | 21 | 267 | -2.74 [-3.82, -1.66] | <0.05 | 85% |
| ≥2 week | 6 | 54 | -1.55 [-2.28, -0.83] | <0.05 | 0% |
| **EVs isolation method** |  |  |  | **<0.05** |  |
| ultracentrifugation | 21 | 241 | -2.53 [-3.62, -1.45] | <0.05 | 84% |
| Commercial kit | 5 | 70 | -2.74 [-3.74, -1.74] | <0.05 | 38% |
| ultrafiltration | 1 | 10 | -0.36 [-1.62, 0.89] | 0.57 | ---- |

**Table S2 Subgroup analysis of the MPO activity**

| **Analysis** | **Trials (n)** | **Analyzes (n)** | **SMD (95% CI)** | **p-value** | **I^2^** |
| --- | --- | --- | --- | --- | --- |
| Overall | 14 | 168 | -2.85 [-3.91, -1.78] | <0.05 | 74% |
| **Animal species** |  |  |  | **<0.05** |  |
| Mice | 11 | 129 | -3.41 [-4.83, -1.99] | <0.05 | 78% |
| Rat | 3 | 39 | -1.56 [-2.59, -0.53] | <0.05 | 27% |
| **Models** |  |  |  | **0.46** |  |
| DSS | 8 | 81 | -2.46 [-3.88, -1.04] | <0.05 | 70% |
| TNBS | 6 | 87 | -3.30 [-5.01, -1.58] | <0.05 | 81% |
| **EVs source** |  |  |  | **<0.05** |  |
| MSCs | 8 | 95 | -2.19 [-3.34, -1.04] | <0.05 | 69% |
| Microbiota | 1 | 20 | -7.66 [-10.45, -4.87] | <0.05 | ---- |
| Milk | 3 | 27 | -2.04 [-3.20, -0.87] | <0.05 | 0% |
| Plants | 2 | 26 | -8.70 [-20.31, 2.91] | 0.14 | 85% |
| **Total doses of EVs** |  |  |  | **0.71** |  |
| ＜200ug | 4 | 52 | -3.74 [-6.95, -0.52] | <0.05 | 87% |
| ≥200ug | 4 | 53 | -3.05 [-4.14, -1.96] | <0.05 | 28% |
| Unknown | 6 | 63 | -2.38 [-4.06, -0.69] | <0.05 | 75% |
| **Time of administration** |  |  |  | **0.81** |  |
| Before colitis induction | 5 | 59 | -3.04 [-4.75, -1.33] | <0.05 | 71% |
| After colitis induction | 9 | 109 | -2.77 [-4.18, -1.35] | <0.05 | 77% |
| **Delivery route** |  |  |  | **0.07** |  |
| intraperitoneal injection | 4 | 50 | -5.47 [-8.78, -2.16] | <0.05 | 78% |
| intravenous injection | 3 | 39 | -1.56 [-2.59, -0.53] | <0.05 | 27% |
| oral gavage | 6 | 63 | -2.38 [-4.06, -0.69] | <0.05 | 75% |
| *in situ* injection | 1 | 16 | -3.40 [-5.08, -1.73] | <0.05 | ---- |
| **Treatment frequency** |  |  |  | **0.15** |  |
| Single administration | 4 | 55 | -2.03 [-3.18, -0.87] | <0.05 | 54% |
| Multiple administration | 10 | 113 | -3.48 [-5.08, -1.88] | <0.05 | 79% |
| **Follow-up duration** |  |  |  | **0.97** |  |
| <2 week | 10 | 131 | -2.89 [-4.17, -1.62] | <0.05 | 78% |
| ≥2week | 4 | 37 | -2.84 [-5.12, -0.57] | <0.05 | 67% |
| **EVs isolation method** |  |  |  | **0.57** |  |
| ultracentrifugation | 12 | 144 | -2.73 [-3.91, -1.55] | <0.05 | 76% |
| Commercial kit | 2 | 24 | -3.67 [-6.71, -0.62] | <0.05 | 71% |

**Table S3 Subgroup analysis of the Histopathological score**

| **Analysis** | **Trials (n)** | **Analyzed (n)** | **SMD (95% CI)** | **p-value** | **I^2^** |
| --- | --- | --- | --- | --- | --- |
| Overall | 10 | 136 | -2.37 [-3.58, -1.16] | <0.05 | 81% |
| **Models** |  |  |  | **0.18** |  |
| DSS | 7 | 80 | -1.90 [-3.16, -0.64] | <0.05 | 75% |
| TNBS | 3 | 56 | -4.36 [-7.71, -1.00] | <0.05 | 90% |
| **EVs resource** |  |  |  | **0.80** |  |
| MSCs | 8 | 110 | -2.55 [-4.06, -1.03] | <0.05 | 85% |
| Microbiota | 2 | 26 | -2.31 [-3.40, -1.22] | <0.05 | 0% |
| **Total doses of EVs** |  |  |  | **<0.05** |  |
| ＜200ug | 2 | 26 | -2.31 [-3.40, -1.22] | <0.05 | 0% |
| ≥200ug | 6 | 90 | -3.80 [-5.87, -1.72] | <0.05 | 86% |
| 3.00E+09 | 1 | 10 | -0.35 [-1.61, 0.90] | 0.58 | ---- |
| Unknown | 1 | 10 | -0.41 [-1.67, 0.85] | 0.52 | ---- |
| **Time of administration** |  |  |  | **0.51** |  |
| Before colitis induction | 3 | 46 | -2.18 [-2.97, -1.40] | <0.05 | 0% |
| After colitis induction | 7 | 90 | -2.87 [-4.73, -1.01] | <0.05 | 87% |
| **Delivery route** |  |  |  | **<0.05** |  |
| intraperitoneal injection | 6 | 90 | -2.28 [-3.51, -1.05] | <0.05 | 74% |
| intravenous injection | 2 | 20 | -1.91 [-4.21, 0.40] | 0.11 | 69% |
| oral gavage | 1 | 16 | -23.98 [-33.60, -14.36] | <0.05 | ---- |
| *in situ* injection | 1 | 10 | -0.41 [-1.67, 0.85] | 0.52 | ---- |
| **Treatment frequency** |  |  |  | **0.15** |  |
| Single administration | 3 | 36 | -6.07 [-11.52, -0.62] | <0.05 | 92% |
| Multiple administration | 7 | 100 | -1.96 [-3.08, -0.84] | <0.05 | 75% |
| **Follow-up duration** |  |  |  | **0.93** |  |
| <2 week | 9 | 130 | -2.41 [-3.71, -1.12] | <0.05 | 83% |
| ≥2 week | 1 | 6 | -2.27 [-4.98, 0.44] | 0.10 | ---- |
| **EVs isolation method** |  |  |  | **<0.05** |  |
| ultracentrifugation | 5 | 66 | -2.58 [-4.70, -0.46] | <0.05 | 86% |
| Commercial kit | 4 | 60 | -3.04 [-4.84, -1.25] | <0.05 | 74% |
| ultrafiltration | 1 | 10 | -0.35 [-1.61, 0.90] | 0.58 | ---- |

**Table S4 Subgroup analysis of the IL-1β**

| **Analysis** | **Trials (n)** | **Analyzed (n)** | **SMD (95% CI)** | **p-value** | **I^2^** |
| --- | --- | --- | --- | --- | --- |
| Overall | 10 | 104 | -1.98 [-2.57, -1.38] | <0.05 | 41% |
| **Animal species** |  |  |  | **0.75** |  |
| Mice | 7 | 65 | -2.06 [-2.83, -1.29] | <0.05 | 37% |
| Rat | 3 | 39 | -1.86 [-2.80, -0.91] | <0.05 | 64% |
| **Models** |  |  |  | **0.95** |  |
| DSS | 6 | 59 | -1.99 [-2.77, -1.22] | <0.05 | 35% |
| TNBS | 4 | 45 | -1.95 [-2.89, -1.02] | <0.05 | 60% |
| **EVs source** |  |  |  | **0.07** |  |
| MSCs | 4 | 49 | -2.05 [-2.91, -1.19] | <0.05 | 54% |
| Microbiota | 1 | 6 | -6.25 [-12.48, -0.01] | 0.05 | ---- |
| Milk | 3 | 27 | -1.16 [-2.16, -0.16] | <0.05 | 0% |
| Macrophages | 1 | 12 | -4.18 [-6.51, -1.84] | <0.05 | ---- |
| Plants | 1 | 10 | -2.84 [-4.87, -0.82] | <0.05 | ---- |
| **Total doses of EVs** |  |  |  | **<0.05** |  |
| ＜200ug | 3 | 32 | -1.53 [-2.55, -0.50] | <0.05 | 43% |
| ≥200ug | 3 | 35 | -3.68 [-4.96, -2.40] | <0.05 | 0% |
| Unknown | 4 | 37 | -1.49 [-2.38, -0.59] | <0.05 | 0% |
| **Time of administration** |  |  |  | **0.47** |  |
| Before colitis induction | 5 | 45 | -1.72 [-2.63, -0.82] | <0.05 | 48% |
| After colitis induction | 5 | 59 | -2.17 [-2.96, -1.38] | <0.05 | 43% |
| **Delivery route** |  |  |  | **0.17** |  |
| Intraperitoneal injection | 1 | 6 | -6.25 [-12.48, -0.01] | 0.05 | ---- |
| Intravenous injection | 5 | 61 | -2.30 [-3.11, -1.49] | <0.05 | 57% |
| Oral gavage | 4 | 37 | -1.49 [-2.38, -0.59] | <0.05 | 0% |
| **Treatment frequency** |  |  |  | **0.83** |  |
| Single administration | 4 | 49 | -2.05 [-2.91, -1.19] | <0.05 | 54% |
| Multiple administration | 6 | 55 | -1.91 [-2.74, -1.08] | <0.05 | 42% |
| **Follow-up duration** |  |  |  | **0.05** |  |
| <2 week | 7 | 77 | -2.43 [-3.17, -1.69] | <0.05 | 46% |
| ≥2week | 3 | 27 | -1.16 [-2.16, -0.16] | <0.05 | 0% |

**Table S5 Subgroup analysis of the IL-10**

| **Analysis** | **Trials (n)** | **Analyzed (n)** | **SMD (95% CI)** | **p-value** | **I^2^** |
| --- | --- | --- | --- | --- | --- |
| Overall | 10 | 97 | 1.78 [1.17, 2.40] | <0.05 | 50% |
| **Animal species** |  |  |  | **0.27** |  |
| Mice | 7 | 58 | 2.58 [1.65, 3.51] | <0.05 | 31% |
| Rat | 3 | 39 | 1.16 [0.33, 1.98] | <0.05 | 60% |
| **Models** |  |  |  | **<0.05** |  |
| DSS | 6 | 52 | 2.52 [1.59, 3.45] | <0.05 | 42% |
| TNBS | 4 | 45 | 1.21 [0.39, 2.03] | <0.05 | 42% |
| **EVs source** |  |  |  | **<0.05** |  |
| MSCs | 5 | 55 | 1.47 [0.72, 2.22] | <0.05 | 29% |
| Microbiota | 1 | 6 | 11.21 [0.27, 22.14] | <0.05 | ---- |
| Milk | 2 | 14 | 1.44 [0.03, 2.86] | 0.05 | 0% |
| Macrophages | 1 | 12 | 6.76 [3.27, 10.24] | <0.05 | ---- |
| Plants | 1 | 10 | 2.70 [0.74, 4.66] | <0.05 | ---- |
| **Total doses of EVs** |  |  |  | **0.10** |  |
| ＜200ug | 4 | 38 | 1.09 [0.15, 2.03] | <0.05 | 52% |
| ≥200ug | 3 | 35 | 2.73 [1.57, 3.89] | <0.05 | 67% |
| Unknown | 3 | 24 | 1.87 [0.73, 3.02] | <0.05 | 0% |
| **Time of administration** |  |  |  | **0.35** |  |
| Before colitis induction | 4 | 32 | 2.32 [1.02, 3.62] | <0.05 | 71% |
| After colitis induction | 6 | 65 | 1.62 [0.93, 2.32] | <0.05 | 28% |
| **Delivery route** |  |  |  | **0.22** |  |
| Intraperitoneal injection | 2 | 12 | 5.38 [1.20, 9.55] | <0.05 | 22% |
| Intravenous injection | 5 | 61 | 1.63 [0.89, 2.37] | <0.05 | 69% |
| Oral gavage | 3 | 24 | 1.87 [0.73, 3.02] | <0.05 | 0% |
| **Treatment frequency** |  |  |  | **0.08** |  |
| Single administration | 4 | 49 | 1.39 [0.63, 2.14] | <0.05 | 25% |
| Multiple administration | 6 | 48 | 2.54 [1.49, 3.60] | <0.05 | 55% |
| **Follow-up duration** |  |  |  | **0.61** |  |
| <2 week | 8 | 83 | 1.86 [1.18, 2.54] | <0.05 | 61% |
| ≥2week | 2 | 14 | 1.44 [0.03, 2.86] | 0.05 | 0% |
