# Supplementary material for: Efficacy of extracellular vesicles as a cell-free therapy in colitis: a systematic review and meta-analysis of animal studies
Source: Front Pharmacol. 2023 Oct 26;14:1260134. doi: 10.3389/fphar.2023.1260134 (PMC10637393; doi:10.3389/fphar.2023.1260134)
Supplement: Supplementary file 3 [file DataSheet1.docx]

**Retrieval strategy**

**PUBMED: 113 records**

#1: ((((((((Colitides[MeSH Terms])OR(Colitis[MeSH Terms])) OR (Colitis, Ischemic[Title/Abstract])) OR (Colitis, Microscopic[Title/Abstract])) OR (Colitis, Collagenous[Title/Abstract])) OR (Colitis, Lymphocytic[Title/Abstract])) OR (Colitis, Ulcerative[Title/Abstract])) OR (Proctocolitis[Title/Abstract]))

#2: ((((((((((((((((((((((((((((((((((Extracellular Vesicles[MeSH Terms]) OR (Extracellular Vesicle[Title/Abstract])) OR (Vesicle, Extracellular[Title/Abstract])) OR (Vesicles, Extracellular[Title/Abstract])) OR (Exovesicles[Title/Abstract])) OR (Exovesicle[Title/Abstract])) OR (Apoptotic Bodies[Title/Abstract])) OR (Apoptotic Body[Title/Abstract])) OR (Bodies, Apoptotic[Title/Abstract])) OR (Body, Apoptotic[Title/Abstract]))OR (Exosomes[MeSH Terms])) OR (Cell-Derived Microparticles[MeSH Terms])) OR (Cell Derived Microparticles[Title/Abstract]))OR (Cell-Derived Microparticle[Title/Abstract])) OR (Microparticle, Cell-Derived[Title/Abstract])) OR (Microparticles, Cell-Derived[Title/Abstract])) OR (Microparticles, Cell Derived[Title/Abstract])) OR (Shedding Microvesicles[Title/Abstract])) OR (Microvesicle, Shedding[Title/Abstract])) OR (Microvesicles, Shedding[Title/Abstract])) OR (Shedding Microvesicle[Title/Abstract])) OR (Cell Membrane Microparticles[Title/Abstract])) OR (Cell Membrane Microparticle[Title/Abstract])) OR (Membrane Microparticle, Cell[Title/Abstract])) OR (Membrane Microparticles, Cell[Title/Abstract])) OR (Microparticle, Cell Membrane[Title/Abstract])) OR (Microparticles, Cell Membrane[Title/Abstract])) OR (Circulating Cell-Derived Microparticles[Title/Abstract])) OR (Cell-Derived Microparticle, Circulating[Title/Abstract])) OR (Cell-Derived Microparticles, Circulating[Title/Abstract])) OR (Circulating Cell Derived Microparticles[Title/Abstract])) OR (Circulating Cell-Derived Microparticle[Title/Abstract])) OR (Microparticle, Circulating Cell-Derived[Title/Abstract])) OR (Microparticles, Circulating Cell-Derived[Title/Abstract]))

#3: #1 AND #2

**EMBASE: 306 records**

#1: 'colitides':ab,ti OR 'colitis':ab,ti OR 'colitis, ischemic':ab,ti OR 'colitis, microscopic':ab,ti OR 'colitis, collagenous':ab,ti OR 'colitis, lymphocytic':ab,ti OR 'colitis, ulcerative':ab,ti OR 'proctocolitis'/exp

#2: 'extracellular vesicle':ab,ti OR 'vesicle, extracellular':ab,ti OR 'vesicles, extracellular':ab,ti OR 'exovesicles':ab,ti OR 'exovesicle':ab,ti OR 'apoptotic bodies':ab,ti OR 'apoptotic body':ab,ti OR 'bodies, apoptotic':ab,ti OR 'body, apoptotic':ab,ti OR 'extracellular vesicles'/exp OR 'exosomes'/exp OR 'cell derived microparticles':ab,ti OR 'cell-derived microparticle':ab,ti OR 'microparticle, cell-derived':ab,ti OR 'microparticles, cell-derived':ab,ti OR 'microparticles, cell derived':ab,ti OR 'shedding microvesicles':ab,ti OR 'microvesicle, shedding':ab,ti OR 'microvesicles, shedding':ab,ti OR 'shedding microvesicle':ab,ti OR 'cell membrane microparticles':ab,ti OR 'cell membrane microparticle':ab,ti OR 'membrane microparticle, cell':ab,ti OR 'membrane microparticles, cell':ab,ti OR 'microparticle, cell membrane':ab,ti OR 'microparticles, cell membrane':ab,ti OR 'circulating cell-derived microparticles':ab,ti OR 'cell-derived microparticle, circulating':ab,ti OR 'cell-derived microparticles, circulating':ab,ti OR 'circulating cell derived microparticles':ab,ti OR 'circulating cell-derived microparticle':ab,ti OR 'microparticle, circulating cell-derived':ab,ti OR 'microparticles, circulating cell-derived':ab,ti OR 'cell-derived microparticles'/exp

#3: #1 AND #2

**Cochrane Library: 3 records**

#1: (Colitides):ti,ab,kw OR (Colitis):ti,ab,kw OR (Colitis, Ischemic):ti,ab,kw OR (Colitis, Microscopic):ti,ab,kw OR (Colitis, Collagenous):ti,ab,kw OR (Colitis, Lymphocytic):ti,ab,kw OR (Colitis, Ulcerative):ti,ab,kw OR (Proctocolitis)

#2: (Extracellular Vesicles):ti,ab,kw OR (Extracellular Vesicle):ti,ab,kw OR (Vesicle, Extracellular):ti,ab,kw OR (Vesicles, Extracellular):ti,ab,kw OR (Exovesicles):ti,ab,kw OR (Exovesicle):ti,ab,kw OR (Apoptotic Bodies):ti,ab,kw OR (Apoptotic Body):ti,ab,kw OR (Bodies, Apoptotic):ti,ab,kw OR (Body, Apoptotic):ti,ab,kw OR (Exosomes):ti,ab,kw OR (Cell-Derived Microparticles):ti,ab,kw OR (Cell Derived Microparticles):ti,ab,kw OR (Cell-Derived Microparticle):ti,ab,kw OR (Microparticle, Cell-Derived):ti,ab,kw OR (Microparticles, Cell-Derived):ti,ab,kw OR (Microparticles, Cell Derived):ti,ab,kw OR (Shedding Microvesicles):ti,ab,kw OR (Microvesicle, Shedding):ti,ab,kw OR (Microvesicles, Shedding):ti,ab,kw OR (Shedding Microvesicle):ti,ab,kw OR (Cell Membrane Microparticles):ti,ab,kw OR (Cell Membrane Microparticle):ti,ab,kw OR (Membrane Microparticle, Cell):ti,ab,kw OR (Membrane Microparticles, Cell):ti,ab,kw OR (Microparticle, Cell Membrane):ti,ab,kw OR (Microparticles, Cell Membrane):ti,ab,kw OR (Circulating Cell-Derived Microparticles):ti,ab,kw OR (Cell-Derived Microparticle, Circulating):ti,ab,kw OR (Cell-Derived Microparticles, Circulating):ti,ab,kw OR (Circulating Cell Derived Microparticles):ti,ab,kw OR (Circulating Cell-Derived Microparticle):ti,ab,kw OR (Microparticle, Circulating Cell-Derived):ti,ab,kw OR (Microparticles, Circulating Cell-Derived)

#3: #1 AND #2

**Web of Science: 345 records**

#1: ((((((((TS=(Colitides) OR TS=(Colitis)) OR TS= (Colitis, Ischemic)) OR TS= (Colitis, Microscopic)) OR TS=(Colitis, Collagenous)) OR TS=(Colitis, Lymphocytic)) OR TS=(Colitis, Ulcerative)) OR TS=(Proctocolitis)))

#2: (((((((((((((((((((((((((((((((((((TS=(Extracellular Vesicles)) OR TS=( Extracellular Vesicle)) OR TS=(Vesicle, Extracellular)) OR TS=(Vesicles, Extracellular)) OR TS=(Exovesicles)) OR TS=(Exovesicle)) OR TS=(Apoptotic Bodies)) OR TS=(Apoptotic Body)) OR TS=(Bodies, Apoptotic)) OR TS=(Body, Apoptotic)) OR TS=(Exosomes)) OR TS=(Cell-Derived Microparticles)) OR TS=(Cell Derived Microparticles)) OR TS=(Cell-Derived Microparticle)) OR TS=(Microparticle, Cell-Derived)) OR TS=(Microparticles, Cell-Derived)) OR TS=(Microparticles, Cell Derived)) OR TS=(Shedding Microvesicles)) OR TS=(Microvesicle, Shedding)) OR TS=(Microvesicles, Shedding)) OR TS=(Shedding Microvesicle)) OR TS=(Cell Membrane Microparticles)) OR TS=(Cell Membrane Microparticle)) OR TS=(Membrane Microparticle, Cell)) OR TS=(Membrane Microparticles, Cell)) OR TS=(Microparticle, Cell Membrane)) OR TS=(Microparticles, Cell Membrane)) OR TS=(Circulating Cell-Derived Microparticles)) OR TS=(Cell-Derived Microparticle, Circulating)) OR TS=(Cell-Derived Microparticles, Circulating)) OR TS=(Circulating Cell Derived Microparticles)) OR TS=(Circulating Cell-Derived Microparticle)) OR TS=(Microparticle, Circulating Cell-Derived)) OR TS=(Microparticles, Circulating Cell-Derived)))

#3: #1 AND #2

**Medline: 78 records**

#1: (SU Colitides OR SU Colitis OR SU Colitis, Ischemic OR SU Colitis, Microscopic OR SU Colitis, Collagenous OR SU Colitis, Lymphocytic OR SU Colitis, Ulcerative OR SU Proctocolitis)

#2: (SU Extracellular Vesicles OR SU Extracellular Vesicle OR SU Vesicle, Extracellular OR SU Vesicles, Extracellular OR SU Exovesicles OR SU Exovesicle OR SU Apoptotic Bodies OR SU Apoptotic Body OR SU Bodies, Apoptotic OR SU Body, Apoptotic OR SU Exosomes OR SU Cell-Derived Microparticles)

#3: (SU Cell Derived Microparticles OR SU Cell-Derived Microparticle OR SU Microparticle, Cell-Derived OR SU Microparticles, Cell-Derived OR SU Microparticles, Cell Derived OR SU Shedding Microvesicles OR SU Microvesicle, Shedding OR SU Microvesicles, Shedding OR SU Shedding Microvesicle OR SU Cell Membrane Microparticles OR SU Cell Membrane Microparticle OR SU Membrane Microparticle, Cell)

#4: (SU Membrane Microparticles, Cell OR SU Microparticle, Cell Membrane OR SU Microparticles, Cell Membrane OR SU Circulating Cell-Derived Microparticles OR SU Cell-Derived Microparticle, Circulating OR SU Cell-Derived Microparticles, Circulating OR SU Circulating Cell Derived Microparticles OR SU Circulating Cell-Derived Microparticle OR SU Microparticle, Circulating Cell-Derived OR SU Microparticles, Circulating Cell-Derived)

#5: (#1) AND (#2 OR #3 OR #4)

**Excluded reason**

A total of 850 pieces of literature were identified for review. We identified 845 records from database searches and 5 additional records from other sources. After removing 311 records due to duplication, we screened 539 records and excluded 424 records based on the title and abstract.

Finally, 115 references were then evaluated through full-text screening, of which 94 references were excluded that 4 for not animal research, 7 due to the animals with comorbidities or defects, 7 due to the non-colitis animal model, 1 because the EVs were not administered to animals directly, 3 because no comparator or inappropriate comparisons, 10 for no relevant outcomes, 2 due to not published in English, and 60 owing to incomplete data. 21 eligible studies were included in our systematic review and meta-analysis. The details for excluded reasons of 94 references are as follows.

1. [1-4] Non-animal research
2. [5-11] Animals with co-morbidities, defects, or custom generated.
3. [12-18]Non colitis animal model
4. [19] EVs are not administered to animals directly.

4. [20-22] No control group or inappropriate comparisons.

5. [23-82] Incomplete or inappropriate data.

6. [83-92] No relevant outcomes or no primary outcomes were reported

7. [93, 94] Not published in English

Reference

1. Alvarez, C.S., et al., *Outer Membrane Vesicles and Soluble Factors Released by Probiotic Escherichia coil Nissle 1917 and Commensal ECOR63 Enhance Barrier Function by Regulating Expression of Tight Junction Proteins in Intestinal Epithelial Cells.* Frontiers in Microbiology, 2016. **7**.

2. Durant, L., et al., *Bacteroides thetaiotaomicron-derived outer membrane vesicles promote regulatory dendritic cell responses in health but not in inflammatory bowel disease.* Microbiome, 2020. **8**(1): p. 88.

3. Mitsuhashi, S., et al., *Luminal Extracellular Vesicles (EVs) in Inflammatory Bowel Disease (IBD) Exhibit Proinflammatory Effects on Epithelial Cells and Macrophages.* Inflammatory Bowel Diseases, 2016. **22**(7): p. 1587-1595.

4. Pak, H., et al., *Safety and efficacy of injection of human placenta mesenchymal stem cells derived exosomes for treatment of complex perianal fistula in non-Crohn's cases: Clinical trial phase I.* Journal of Gastroenterology and Hepatology, 2023. **38**(4): p. 539-547.

5. Bauer, K.M., et al., *CD11c+ myeloid cell exosomes reduce intestinal inflammation during colitis.* JCI Insight, 2022. **7**(19).

6. Chen, H., et al., *Extracellular Vesicles from Apoptotic Cells Promote TGFβ Production in Macrophages and Suppress Experimental Colitis.* Sci Rep, 2019. **9**(1): p. 5875.

7. Golan-Gerstl, R., et al., *Milk derived exosomes has a therapeutic effect on experimental colitis.* Journal of Crohns & Colitis, 2020. **14**: p. S155-S155.

8. Jiang, L.L., et al., *EpCAM-dependent extracellular vesicles from intestinal epithelial cells maintain intestinal tract immune balance.* Nature Communications, 2016. **7**.

9. Stremmel, W., R. Weiskirchen, and B.C. Melnik, *Milk Exosomes Prevent Intestinal Inflammation in a Genetic Mouse Model of Ulcerative Colitis: A Pilot Experiment.* Inflammatory Intestinal Diseases, 2020. **5**(3): p. 117-123.

10. Wei, S., et al., *Fusobacterium nucleatum Extracellular Vesicles Promote Experimental Colitis by Modulating Autophagy via the miR-574-5p/CARD3 Axis.* Inflamm Bowel Dis, 2023. **29**(1): p. 9-26.

11. Wu, D., et al., *Dietary Depletion of Milk Exosomes and Their MicroRNA Cargos Elicits a Depletion of miR-200a-3p and Elevated Intestinal Inflammation and Chemokine (C-X-C Motif) Ligand 9 Expression in Mdr1a(-/-) Mice.* Current Developments in Nutrition, 2019. **3**(12).

12. Sun, D., et al., *MiR-200b in heme oxygenase-1-modified bone marrow mesenchymal stem cell-derived exosomes alleviates inflammatory injury of intestinal epithelial cells by targeting high mobility group box 3.* Cell Death & Disease, 2020. **11**(6).

13. Barry, M., et al., *Mesenchymal stem cell extracellular vesicles mitigate vascular permeability and injury in the small intestine and lung in a mouse model of hemorrhagic shock and trauma.* Journal of Trauma and Acute Care Surgery, 2022. **92**(3): p. 489-498.

14. Chu, S., et al., *Exosomes derived from EphB2-overexpressing bone marrow mesenchymal stem cells regulate immune balance and repair barrier function.* Biotechnol Lett, 2023. **45**(5-6): p. 601-617.

15. Izco, M., et al., *Targeted Extracellular Vesicle Gene Therapy for Modulating Alpha-Synuclein Expression in Gut and Spinal Cord.* Pharmaceutics, 2023. **15**(4).

16. Shao, Y., et al., *Circulating exosomal miR-155-5p contributes to severe acute pancreatitis-associated intestinal barrier injury by targeting SOCS1 to activate NLRP3 inflammasome-mediated pyroptosis.* Faseb Journal, 2023. **37**(6).

17. Wang, J., et al., *hucMSC-Derived Exosomes Alleviate the Deterioration of Colitis via the miR-146a/SUMO1 Axis.* Mol Pharm, 2022. **19**(2): p. 484-493.

18. Yaghoubfar, R., et al., *Modulation of serotonin signaling/metabolism by Akkermansia muciniphila and its extracellular vesicles through the gut-brain axis in mice.* Scientific Reports, 2020. **10**(1).

19. Zhao, F., et al., *Extracellular vesicles package dsDNA to aggravate Crohn's disease by activating the STING pathway.* Cell Death Dis, 2021. **12**(9): p. 815.

20. Chen, D., et al., *High-fat diet aggravates colitis via mesenteric adipose tissue derived exosome metastasis-associated lung adenocarcinoma transcript 1.* World J Gastroenterol, 2022. **28**(29): p. 3838-3853.

21. Joo, H., et al., *Extracellular Vesicles from Thapsigargin-Treated Mesenchymal Stem Cells Ameliorated Experimental Colitis via Enhanced Immunomodulatory Properties.* Biomedicines, 2021. **9**(2).

22. Li, P., et al., *Mesenchymal stem cells-derived extracellular vesicles containing miR-378a-3p inhibit the occurrence of inflammatory bowel disease by targeting GATA2.* Journal of Cellular and Molecular Medicine, 2022. **26**(11): p. 3133-3146.

23. Xu, F., et al., *Mesenchymal Stem Cell-Derived Extracellular Vesicles with High PD-L1 Expression for Autoimmune Diseases Treatment.* Advanced Materials, 2022. **34**(1).

24. Barnhoorn, M., et al., *Mesenchymal stromal cell-derived exosomes stimulate epithelial regeneration in vitro and reduce experimental colitis.* Journal of Crohns & Colitis, 2019. **13**: p. S15-S15.

25. Bray, G. and P. Giacomin, *Evaluation of helminth extracellular vesicles as a novel therapeutic for inflammatory bowel disease in murine models of colitis.* Journal of Crohns & Colitis, 2022. **16**: p. I607-I608.

26. Bulut, E.A., et al., *Human Gut Commensal Membrane Vesicles Modulate Inflammation by Generating M2-like Macrophages and Myeloid-Derived Suppressor Cells.* Journal of Immunology, 2020. **205**(10): p. 2707-2718.

27. Chang, C.L., et al., *Synergistic effect of combined melatonin and adipose-derived mesenchymal stem cell (ADMSC)-derived exosomes on amelioration of dextran sulfate sodium (DSS)-induced acute colitis.* American Journal of Translational Research, 2019. **11**(5): p. 2706-2724.

28. Deng, F., et al., *M2 Macrophage-Derived Exosomal miR-590-3p Attenuates DSS-Induced Mucosal Damage and Promotes Epithelial Repair via the LATS1/YAP/ β-Catenin Signalling Axis.* J Crohns Colitis, 2021. **15**(4): p. 665-677.

29. Du, C.M., et al., *Bovine milk-derived extracellular vesicles prevent gut inflammation by regulating lipid and amino acid metabolism.* Food & Function, 2023. **14**(4): p. 2212-2222.

30. Feng, T., et al., *EXOSOMES DERIVED FROM HUMAN IPSC-MSCS PROTECTED AGAINST TNBS-INDUCED COLITIS.* Gut, 2018. **67**: p. A12-A12.

31. Fonseca, S., et al., *Extracellular vesicles produced by the human gut commensal bacterium Bacteroides thetaiotaomicron elicit anti-inflammatory responses from innate immune cells.* Frontiers in Microbiology, 2022. **13**.

32. Gao, H.N., et al., *Yak milk-derived exosomes alleviate lipopolysaccharide-induced intestinal inflammation by inhibiting PI3K/AKT/C3 pathway activation.* Journal of Dairy Science, 2021. **104**(8): p. 8411-8424.

33. Giner, R.M., et al., *EXTRACELLULAR VESICLES FROM FASCIOLA HEPATICA ADULTS ATTENUATES MUCOSAL INTESTINAL DAMAGE IN DEXTRAN SULFATE SODIUM INDUCED COLITIS IN MICE.* Basic & Clinical Pharmacology & Toxicology, 2015. **117**: p. 6-6.

34. Gong, L.Q., et al., *Immunomodulatory Effect of Serum Exosomes From Crohn Disease on Macrophages via Let-7b-5p/TLR4 Signaling.* Inflammatory Bowel Diseases, 2022. **28**(1): p. 96-108.

35. Gu, L., et al., *Exosomal MicroRNA-181a Derived From Mesenchymal Stem Cells Improves Gut Microbiota Composition, Barrier Function, and Inflammatory Status in an Experimental Colitis Model.* Frontiers in Medicine, 2021. **8**.

36. Hao, H., et al., *Effect of Extracellular Vesicles Derived From Lactobacillus plantarum Q7 on Gut Microbiota and Ulcerative Colitis in Mice.* Front Immunol, 2021. **12**: p. 777147.

37. He, H.X., et al., *Extracellular vesicles produced by bone marrow mesenchymal stem cells overexpressing programmed death-ligand 1 ameliorate dextran sodium sulfate-induced ulcerative colitis in rats by regulating Th17/Treg cell balance through PTEN/PI3K/AKT/mTOR axis.* Journal of Gastroenterology and Hepatology, 2022. **37**(12): p. 2243-2254.

38. Jie, Z., et al., *Immunosuppressive Effect of Exosomes from Mesenchymal Stromal Cells in Defined Medium on Experimental Colitis.* International Journal of Stem Cells, 2019. **12**(3): p. 440-448.

39. Kang, E.A., et al., *Extracellular Vesicles Derived from Kefir Grain Lactobacillus Ameliorate Intestinal Inflammation via Regulation of Proinflammatory Pathway and Tight Junction Integrity.* Biomedicines, 2020. **8**(11).

40. Kim, J., et al., *Amelioration of colitis progression by ginseng-derived exosome-like nanoparticles through suppression of inflammatory cytokines.* Journal of Ginseng Research, 2023.

41. Liu, H.S., et al., *Exosomes from mesenchymal stromal cells reduce murine colonic inflammation via a macrophage-dependent mechanism.* Jci Insight, 2019. **4**(24).

42. Ma, L., et al., *Anti-Inflammatory Effect of Clostridium butyricum-Derived Extracellular Vesicles in Ulcerative Colitis: Impact on Host microRNAs Expressions and Gut Microbiome Profiles.* Mol Nutr Food Res, 2023: p. e2200884.

43. Ma, Z.J., et al., *Immunosuppressive effect of exosomes from mesenchymal stromal cells in defined medium on experimental colitis.* International Journal of Stem Cells, 2019. **12**(3): p. 440-448.

44. Mao, F., et al., *Exosomes Derived from Human Umbilical Cord Mesenchymal Stem Cells Relieve Inflammatory Bowel Disease in Mice.* Biomed Research International, 2017. **2017**.

45. Reif, S., et al., *Cow and Human Milk-Derived Exosomes Ameliorate Colitis in DSS Murine Model.* Nutrients, 2020. **12**(9).

46. Roig, J., et al., *Extracellular Vesicles From the Helminth Fasciola hepatica Prevent DSS-Induced Acute Ulcerative Colitis in a T-Lymphocyte Independent Mode.* Frontiers in Microbiology, 2018. **9**.

47. Shen, Q., et al., *Extracellular vesicle miRNAs promote the intestinal microenvironment by interacting with microbes in colitis.* Gut Microbes, 2022. **14**(1): p. 2128604.

48. Wang, L.F., et al., *Exosomes Derived from Dendritic Cells Treated with Schistosoma japonicum Soluble Egg Antigen Attenuate DSS-Induced Colitis.* Frontiers in Pharmacology, 2017. **8**.

49. Wei, Z.P., et al., *Human umbilical cord mesenchymal stem cells derived exosome shuttling mir-129-5p attenuates inflammatory bowel disease by inhibiting ferroptosis.* Journal of Nanobiotechnology, 2023. **21**(1).

50. Xu, Y.T., et al., *HucMSC-Ex carrying miR-203a-3p.2 ameliorates colitis through the suppression of caspase11/4-induced macrophage pyroptosis.* International Immunopharmacology, 2022. **110**.

51. Yang, S., et al., *Exosomes derived from human umbilical cord mesenchymal stem cells to attenuate colitis through repairing intestinal mucosal barrier.* Journal of Crohns & Colitis, 2021. **15**: p. S105-S105.

52. Zhang, L., et al., *Exosomes derived from human umbilical cord mesenchymal stem cells regulate lymphangiogenesis via the miR-302d-3p/VEGFR3/AKT axis to ameliorate inflammatory bowel disease.* International Immunopharmacology, 2022. **110**.

53. Zhang, Y., et al., *Exosomes derived from 3D-cultured MSCs improve therapeutic effects in periodontitis and experimental colitis and restore the Th17 cell/Treg balance in inflamed periodontium.* Int J Oral Sci, 2021. **13**(1): p. 43.

54. Zhu, Y.L., et al., *Endometrial Regenerative Cell-Derived Exosomes Attenuate Experimental Colitis through Downregulation of Intestine Ferroptosis.* Stem Cells International, 2022. **2022**.

55. Appiah, M.G., et al., *Intestinal Epithelium-Derived Luminally Released Extracellular Vesicles in Sepsis Exhibit the Ability to Suppress TNF-a and IL-17A Expression in Mucosal Inflammation.* Int J Mol Sci, 2020. **21**(22).

56. Benmoussa, A., et al., *Concentrates of two subsets of extracellular vesicles from cow's milk modulate symptoms and inflammation in experimental colitis.* Sci Rep, 2019. **9**(1): p. 14661.

57. Yang, X., et al., *Exosomes derived from interleukin-10-treated dendritic cells can inhibit trinitrobenzene sulfonic acid-induced rat colitis.* Scand J Gastroenterol, 2010. **45**(10): p. 1168-77.

58. An, J.H., et al., *TSG-6 in extracellular vesicles from canine mesenchymal stem/stromal is a major factor in relieving DSS-induced colitis.* PLoS One, 2020. **15**(2): p. e0220756.

59. Cai, X., et al., *hucMSC-derived exosomes attenuate colitis by regulating macrophage pyroptosis via the miR-378a-5p/NLRP3 axis.* Stem Cell Res Ther, 2021. **12**(1): p. 416.

60. Chang, Y., et al., *From Hair to Colon: Hair Follicle-Derived MSCs Alleviate Pyroptosis in DSS-Induced Ulcerative Colitis by Releasing Exosomes in a Paracrine Manner.* Oxid Med Cell Longev, 2022. **2022**: p. 9097530.

61. Chen, Q., et al., *BMSC-EVs regulate Th17 cell differentiation in UC via H3K27me3.* Mol Immunol, 2020. **118**: p. 191-200.

62. Yan, Y., et al., *Perinatal tissue-derived exosomes ameliorate colitis in mice by regulating the Foxp3 + Treg cells and gut microbiota.* Stem Cell Res Ther, 2023. **14**(1): p. 43.

63. Dadgar, N., et al., *Effect of Crohn's disease mesenteric mesenchymal stem cells and their extracellular vesicles on T-cell immunosuppressive capacity.* J Cell Mol Med, 2022. **26**(19): p. 4924-4939.

64. Deng, C., et al., *Oral delivery of layer-by-layer coated exosomes for colitis therapy.* J Control Release, 2023. **354**: p. 635-650.

65. Du, C., et al., *Effects of Milk-Derived Extracellular Vesicles on the Colonic Transcriptome and Proteome in Murine Model.* Nutrients, 2022. **14**(15).

66. Eichenberger, R.M., et al., *Hookworm Secreted Extracellular Vesicles Interact With Host Cells and Prevent Inducible Colitis in Mice.* Front Immunol, 2018. **9**: p. 850.

67. Gao, X., et al., *Extracellular vesicles derived from Trichinella spiralis prevent colitis by inhibiting M1 macrophage polarization.* Acta Trop, 2021. **213**: p. 105761.

68. Han, G., et al., *Bovine colostrum derived-exosomes prevent dextran sulfate sodium-induced intestinal colitis via suppression of inflammation and oxidative stress.* Biomater Sci, 2022. **10**(8): p. 2076-2087.

69. Heidari, N., et al., *Adipose-derived mesenchymal stem cell-secreted exosome alleviates dextran sulfate sodium-induced acute colitis by Treg cell induction and inflammatory cytokine reduction.* Journal of Cellular Physiology, 2021. **236**(8): p. 5906-5920.

70. Li, Y., J. Altemus, and A.L. Lightner, *Mesenchymal stem cells and acellular products attenuate murine induced colitis.* Stem Cell Res Ther, 2020. **11**(1): p. 515.

71. Ma, L., et al., *Clostridium butyricum and Its Derived Extracellular Vesicles Modulate Gut Homeostasis and Ameliorate Acute Experimental Colitis.* Microbiol Spectr, 2022. **10**(4): p. e0136822.

72. Ocansey, D.K.W., et al., *Mesenchymal stem cell-derived exosome mitigates colitis via the modulation of the gut metagenomics-metabolomics-farnesoid X receptor axis.* Biomater Sci, 2022. **10**(17): p. 4822-4836.

73. Tian, J., et al., *Olfactory Ecto-Mesenchymal Stem Cell-Derived Exosomes Ameliorate Experimental Colitis via Modulating Th1/Th17 and Treg Cell Responses.* Front Immunol, 2020. **11**: p. 598322.

74. Wang, G., et al., *HucMSC-exosomes carrying miR-326 inhibit neddylation to relieve inflammatory bowel disease in mice.* Clin Transl Med, 2020. **10**(2): p. e113.

75. Wang, X., et al., *Versatility of bacterial outer membrane vesicles in regulating intestinal homeostasis.* Sci Adv, 2023. **9**(11): p. eade5079.

76. Wang, Y., et al., *Exosomes released by granulocytic myeloid-derived suppressor cells attenuate DSS-induced colitis in mice.* Oncotarget, 2016. **7**(13): p. 15356-68.

77. Wu, H., et al., *Extracellular vesicles containing miR-146a attenuate experimental colitis by targeting TRAF6 and IRAK1.* Int Immunopharmacol, 2019. **68**: p. 204-212.

78. Xu, H.K., et al., *TNF-alpha Enhances the Therapeutic Effects of MenSC-Derived Small Extracellular Vesicles on Inflammatory Bowel Disease through Macrophage Polarization by miR-24-3p.* Stem Cells International, 2023. **2023**.

79. Yang, R., et al., *Exosomes Derived From M2b Macrophages Attenuate DSS-Induced Colitis.* Front Immunol, 2019. **10**: p. 2346.

80. Youn, Y.J., et al., *Neutrophil-derived trail is a proinflammatory subtype of neutrophil-derived extracellular vesicles.* Theranostics, 2021. **11**(6): p. 2770-2787.

81. Yu, T., et al., *Extracellular vesicles derived from EphB2-overexpressing bone marrow mesenchymal stem cells ameliorate DSS-induced colitis by modulating immune balance.* Stem Cell Res Ther, 2021. **12**(1): p. 181.

82. Zhang, Y., et al., *Immune modulation mediated by extracellular vesicles of intestinal organoids is disrupted by opioids.* Mucosal Immunol, 2021. **14**(4): p. 887-898.

83. Barnhoorn, M.C., et al., *Mesenchymal Stromal Cell-Derived Exosomes Contribute to Epithelial Regeneration in Experimental Inflammatory Bowel Disease.* Cell Mol Gastroenterol Hepatol, 2020. **9**(4): p. 715-717.e8.

84. Chen, Y.F., et al., *Serum exosomes derived from Hp-positive gastritis patients inhibit MCP-1 and MIP-1 alpha expression via NLRP12-Notch signaling pathway in intestinal epithelial cells and improve DSS-induced colitis in mice.* International Immunopharmacology, 2020. **88**.

85. Du, C., et al., *Supplementation with Milk-Derived Extracellular Vesicles Shapes the Gut Microbiota and Regulates the Transcriptomic Landscape in Experimental Colitis.* Nutrients, 2022. **14**(9).

86. El-Desoky Mohamady, R.E., et al., *Effect of mesenchymal stem cells derived exosomes and green tea polyphenols on acetic acid induced ulcerative colitis in adult male albino rats.* Ultrastruct Pathol, 2022. **46**(2): p. 147-163.

87. Gao, X., et al., *Extracellular vesicles from Trichinella spiralis: Proteomic analysis and protective immunity.* Plos Neglected Tropical Diseases, 2022. **16**(6).

88. Guo, J., et al., *Exosome-based bone-targeting drug delivery alleviates impaired osteoblastic bone formation and bone loss in inflammatory bowel diseases.* Cell Rep Med, 2023. **4**(1): p. 100881.

89. Kim, S.H., et al., *Immunomodulatory Activity of Extracellular Vesicles of Kimchi-Derived Lactic Acid Bacteria (Leuconostoc mesenteroides, Latilactobacillus curvatus, and Lactiplantibacillus plantarum).* Foods, 2022. **11**(3).

90. Liao, F., X.H. Lu, and W.G. Dong, *Exosomes derived from T regulatory cells relieve inflammatory bowel disease by transferring miR-195a-3p.* Iubmb Life, 2020. **72**(12): p. 2591-2600.

91. Yang, J., et al., *miR-200b-containing microvesicles attenuate experimental colitis associated intestinal fibrosis by inhibiting epithelial-mesenchymal transition.* J Gastroenterol Hepatol, 2017. **32**(12): p. 1966-1974.

92. Yu, H.L., et al., *Human Adipose Mesenchymal Stem Cell-derived Exosomes Protect Mice from DSS-Induced Inflammatory Bowel Disease by Promoting Intestinal-stem-cell and Epithelial Regeneration.* Aging and Disease, 2021. **12**(6): p. 1423-1437.

93. Chen, S.Z. and Y. Chen, *[The role of intestinal bacteria derived extracellular vesicles in inflammatory bowel disease].* Zhonghua Nei Ke Za Zhi, 2021. **60**(10): p. 932-936.

94. Duan, L. and X. Cao, *Human placenta mesenchymal stem cells-derived extracellular vesicles regulate collagen deposition in intestinal mucosa of mice with colitis.* Chinese Journal of Tissue Engineering Research, 2020. **25**(7): p. 1026-1031.
